# Autocatalytic Activation of a Ruthenium-PNN-Pincer Hydrogenation Catalyst

**DOI:** 10.1021/acscatal.4c04475

**Published:** 2024-10-23

**Authors:** Jose Fernando Carbajal Perez, Fallyn L. Kirlin, Eamon F. Reynolds, Cole E. Altomare-Jarczyk, Benjamin T. Joseph, Jason M. Keith, Anthony R. Chianese

**Affiliations:** Department of Chemistry, Colgate University, 13 Oak Drive, Hamilton, New York 13346, United States

**Keywords:** autocatalysis, DFT, kinetics, mechanism, hydrogenation

## Abstract

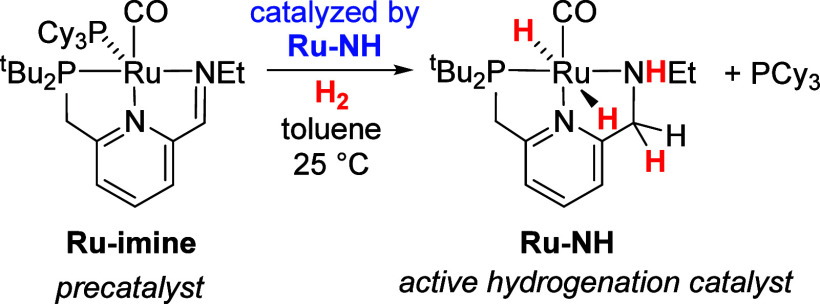

In this article, we describe a detailed experimental and computational study of the activation mechanism for a highly active pincer ruthenium(0) precatalyst for the hydrogenation of polar organic compounds. The precatalyst activates by reaction with 2 equiv of hydrogen, resulting in a net oxidative addition to ruthenium and hydrogenation of an imine functional group on the supporting ligand. The kinetics of precatalyst hydrogenation were measured by UV–visible spectroscopy under catalytically relevant conditions (10–39 bar hydrogen, 298 K). The kinetic data, in combination with density functional theory calculations, support an intriguing autocatalytic mechanism, where the product ruthenium(II) complex catalyzes the hydrogenation of the ruthenium(0) precatalyst.

## Introduction

**Scheme 1:**
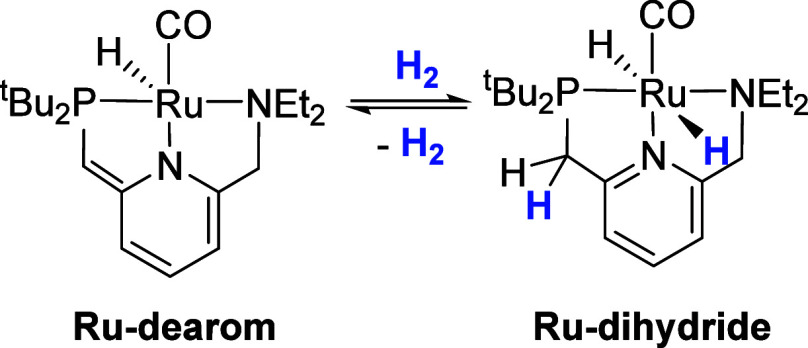
Reversible Addition of H_2_ to Milstein’s Catalyst, **Ru-dearom**

The development of hydrogenation and dehydrogenation catalysts benefitting from metal–ligand cooperativity, spurred initially by the groups of Noyori^1^ and Shvo,^2^ was substantially advanced with the discovery by Milstein and co-workers of a highly active ruthenium-pincer catalyst for ester hydrogenation^3^ and the reverse reaction, acceptorless dehydrogenation of primary alcohols.^4^ The Milstein catalyst was subsequently applied to the hydrogenation of carbonate esters^5^ and carbon dioxide,^6^ as well as dehydrogenative couplings to form amides^7^ and conjugated imines.^8^ Milstein’s catalyst **Ru-dearom** rapidly and reversibly reacts with H_2_ to form **Ru-dihydride**, formally the result of protonation of the pincer ligand at carbon and hydride addition to ruthenium (Scheme 1). This novel metal–ligand-cooperative behavior was proposed to occur as a key step in hydrogenation^3,9^ and the intermediacy of **Ru-dearom** and/or **Ru-H** featured prominently in DFT studies of Milstein’s catalyst.^10^

**Scheme 2:**
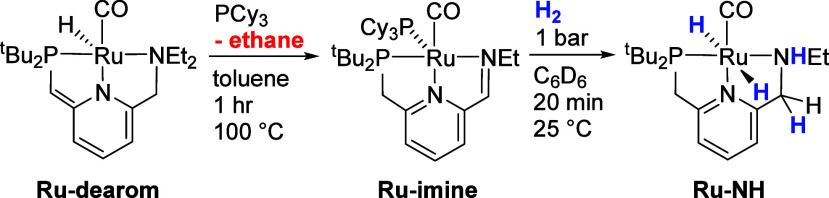
Conversion of **Ru-dearom** to **Ru-NH** by Ethane Release Followed by Reaction with H_2_

In 2019, we demonstrated experimentally that **Ru-dearom** and **Ru-dihydride** are precatalysts rather than active intermediates in ester hydrogenation catalysis.^11^ When heated, **Ru-dearom** releases an equivalent of ethane, giving a ruthenium(0) intermediate that can be trapped with PCy_3_ to form the five-coordinate pincer complex **Ru-imine** (Scheme 2, left). **Ru-imine** is the most active additive-free ester-hydrogenation catalyst known, giving in excess of 10,000 turnovers at room temperature for a range of ester substates. In this prior study, we showed through NMR spectroscopy that **Ru-imine** activates at room temperature by reaction with two equivalents of H_2_ to give the Noyori-type dihydride complex **Ru-NH** (Scheme 2, right). In a subsequent combined experimental/computational study, we obtained a crystal structure verifying the structure of **Ru-NH**, and showed that the nascent N-H group in **Ru-NH** is essential for catalytic ester hydrogenation, calling into question the involvement of CH_2_ linkers in reactions catalyzed by **Ru-dearom**.^12^

The hydrogenation of an imine or pyridine functionality on a supporting ligand has been demonstrated for several closely related catalyst systems, as shown in 1. Schneider and co-workers observed the net hydrogenation of an ene-amido fragment in a PNP-Ru complex, and proposed a mechanism involving metal–ligand-cooperative addition of H_2_ to Ru and a ligand carbon, giving an intermediate with an imine group on the ligand, followed by hydride migration from Ru to the imine carbon.^13^ Santiso-Quinones, Grützmacher, and co-workers synthesized a Ru complex containing a tripodal alkene-amine-pyridine ligand by hydrogenation of the analogous alkene-imine-pyridine ligand, in the presence of KO^t^Bu and H_2_.^14^ Braun and co-workers observed the hydrogenation of a C = N unit in a diimine-iridium complex.^15^ Saito and workers demonstrated that hydrogenation of the pyridine units in a Ru(PN)_2_Cl_2_ precatalyst was a necessary step in catalyst activation for amide hydrogenation,^16^ as well as in a related Ir(PNNP)HCl catalyst for carboxylic acid hydrogenation.^17^ Khaskin, Gusev, and co-workers showed that a PNN-Ru precatalyst similarly was activated by hydrogenation of a pyridine ring in the ligand.^18^

**Chart 1:**
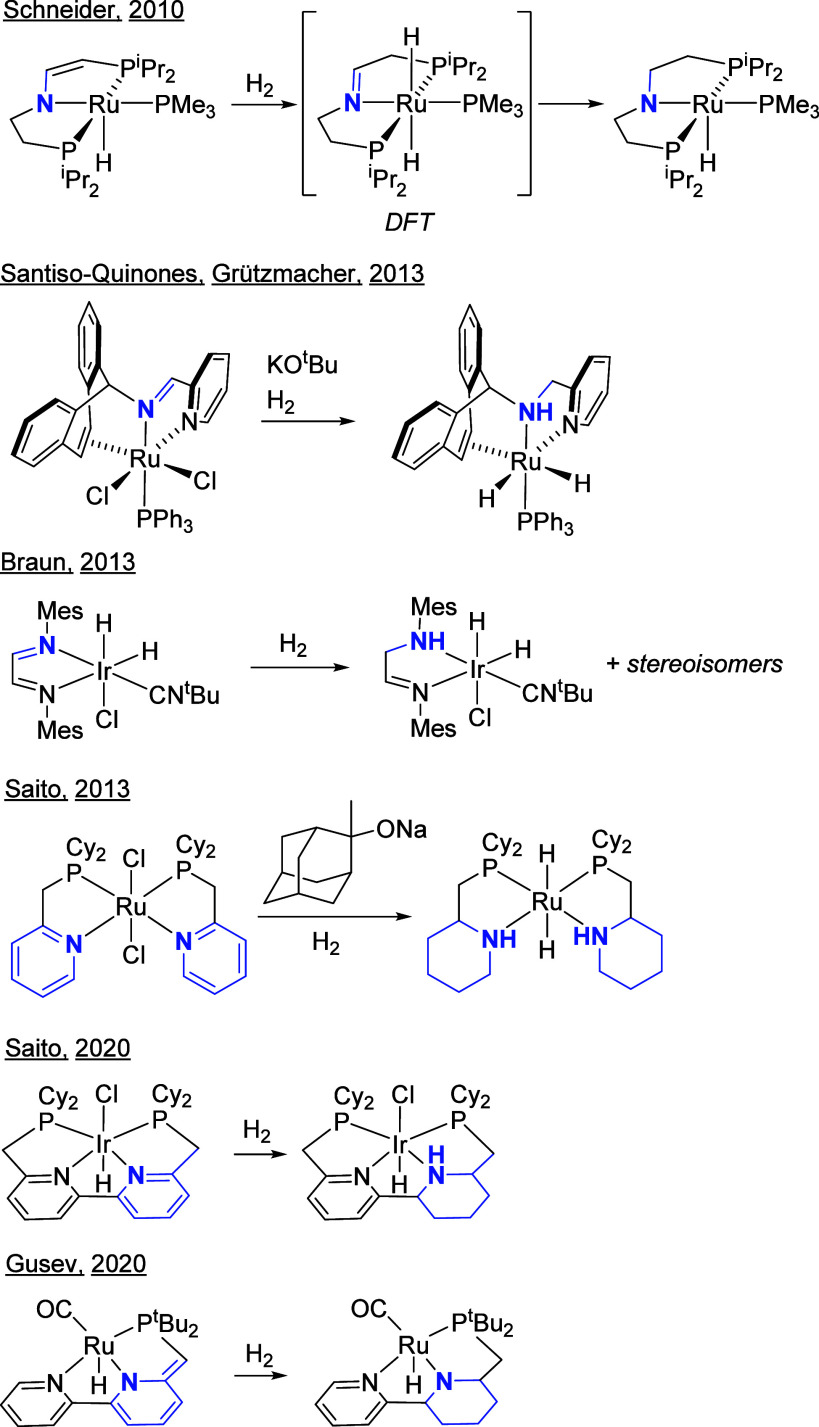
Hydrogenation Catalysts Featuring Hydrogenation of an Imine or Pyridine Functional Group on the Supporting Ligand

The conversion of **Ru-imine** to **Ru-NH** (Scheme 2, right) involves the release of PCy_3_ and the incorporation of two equivalents of H_2_, resulting in a net oxidative addition to Ru and hydrogenation of the ligand’s C = N double bond. Because of the complexity of this transformation and its centrality in the activation of the widely used Milstein catalyst, we initiated a combined experimental/computational study of its mechanism. The kinetic and computational data support an interesting and unexpected autocatalytic pathway, where **Ru-NH** catalyzes the hydrogenation of the C = N double bond in **Ru-imine**, resulting in sigmoidal kinetics. This study provides insight into the specific activation mechanism of this catalyst, but may have broader relevance in hydrogenation catalysis, due to the ubiquity of ligand hydrogenation in catalyst activation as described above.

## Kinetic Studies

### Initial Measurements at 1 Bar H_2_

**Figure 1:**
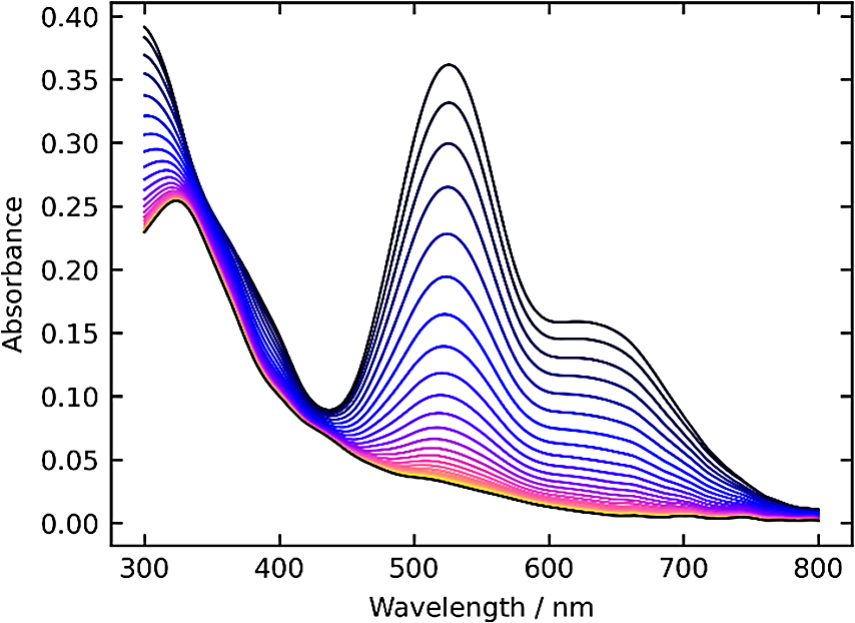
Evolution of the UV–vis spectrum of **Ru-imine** under 1 bar H_2_ at room temperature.

Kinetic analysis of the double hydrogenation of **Ru-imine** to give **Ru-NH** requires monitoring the time course of the reaction under varying pressures of hydrogen gas. Because the reactant **Ru-imine** is intensely purple and the product **Ru-NH** is pale yellow, we decided to monitor the reaction by UV–visible spectroscopy. Figure 1 shows the evolution of the absorption spectrum of a 0.050 mM solution of **Ru-imine** in toluene under 1 bar H_2_ at room temperature, monitored for one hour. As **Ru-imine** converts to **Ru-NH**, two absorbance features in the visible spectrum, centered at 530 and 650 nm, decrease in intensity. No isosbestic points were observable, as the absorptivity of **Ru-imine** is greater than that of **Ru-NH** across the spectrum.

**Scheme 3:**
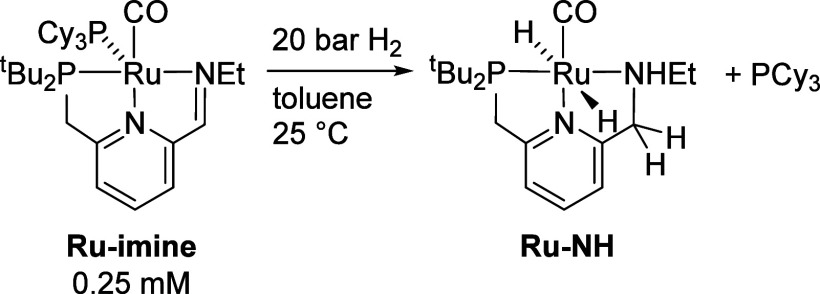
Standard Conditions for Kinetic Experiments

To monitor this transformation under catalytically relevant pressures of H_2_, we developed a modified stainless steel pressure reactor, fitted with fused-silica windows to allow observation in the UV–vis spectral range. To allow quantitation of [**Ru-imine**] at the higher concentrations necessary to observe autocatalytic behavior (see below), the absorbance was tracked at 700 nm rather than the λ_max_ of 530 nm. A standard series (Figure S1) confirmed the linearity of the response at 700 nm. The standard reaction conditions are shown in Scheme 3. To determine the effects of the reactant and product concentrations on the rate, we conducted experiments where the initial concentrations of **Ru-imine** and PCy_3_, as well as the hydrogen pressure, were systematically varied from the standard conditions.

### Variation of **[Ru-imine]_0_**

**Figure 2:**
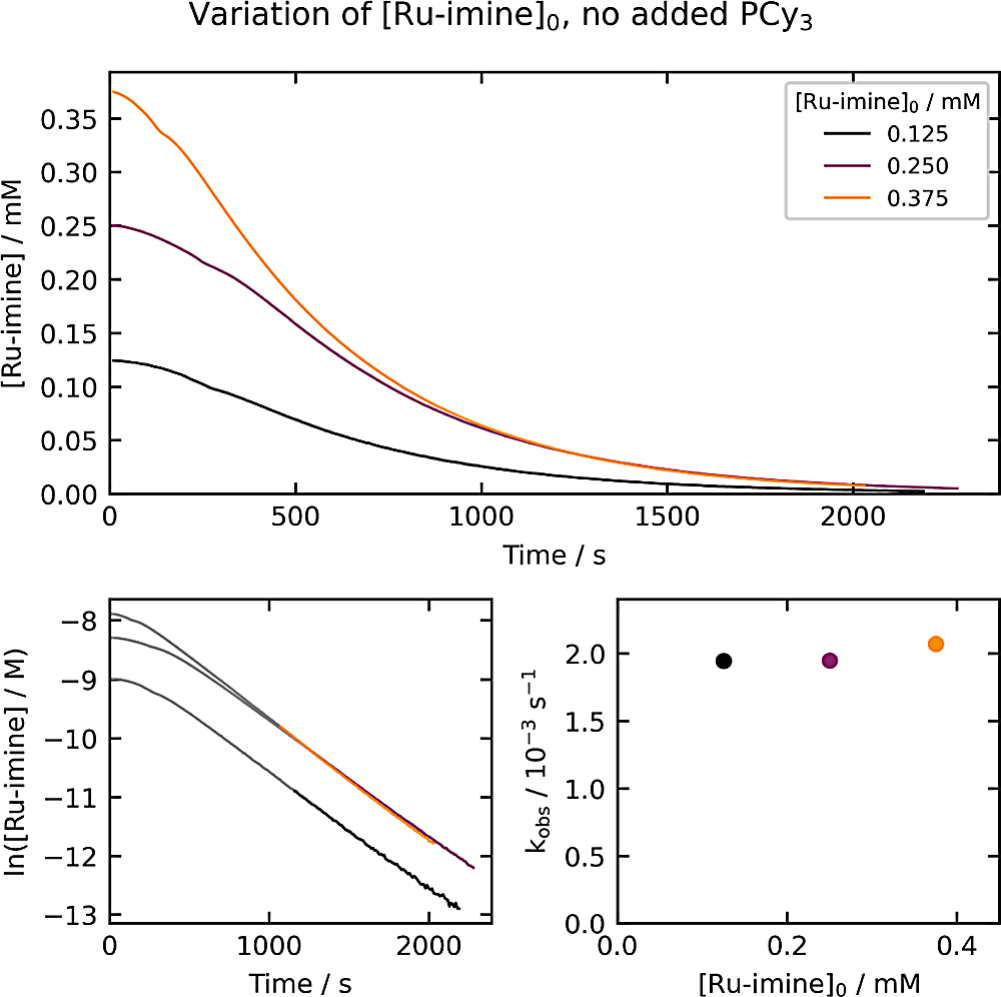
Kinetics of **Ru-imine** hydrogenation with varying [**Ru-imine**]_0_. The top plot shows the evolution of [**Ru-imine**] over time, and the bottom-left plot shows the evolution of ln[**Ru-imine**] over time. The bottom-right plot shows the dependence of k_obs_ on [**Ru-imine**]_0_. The k_obs_ values (top right) are derived from the period between 85% and 98% conversion, as highlighted in the bottom-left plots.

First, we conducted three experiments where the initial concentration of **Ru-imine** was varied from 0.125 mM to 0.375 mM (Figure 2). In each experiment, an initial acceleration period lasts for 3–5 min, and may be associated with mass transfer of hydrogen gas into solution. Following this, the reaction displays pseudo-first-order kinetics, as indicated by the linearity of the plot of ln[**Ru-imine**] vs time. The pseudo-first-order k_obs_ value is constant as [**Ru-imine**]_0_ is varied, suggesting that saturation in [**Ru-imine**] and any effect of the product **Ru-NH** are minimal under these conditions.

### Variation of the Hydrogen Pressure

**Figure 3:**
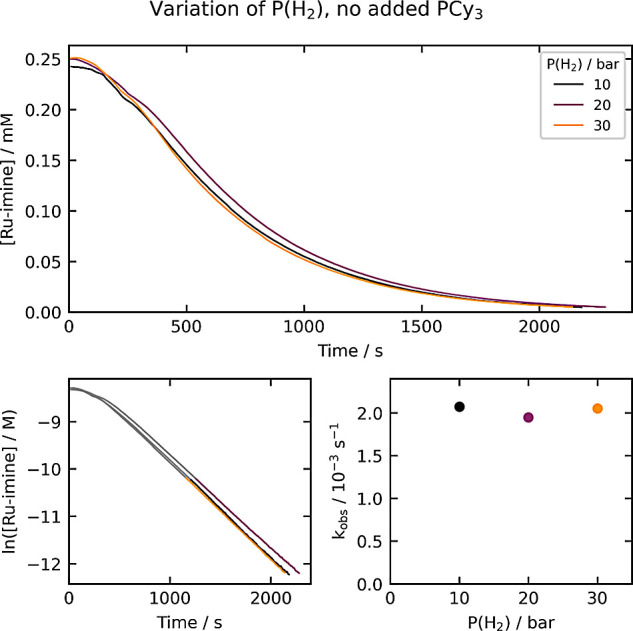
Kinetics of **Ru-imine** hydrogenation under varying hydrogen pressure. The top plot shows the evolution of [**Ru-imine**] over time, and the bottom-left plot shows the evolution of ln[**Ru-imine**] over time. The bottom-right plot shows the dependence of k_obs_ on the hydrogen pressure. The k_obs_ values (top right) are derived from the period between 85% and 98% conversion, as highlighted in the bottom-left plots.

Next, we conducted experiments where the hydrogen pressure, maintained as a constant throughout each reaction, was varied from 10 to 30 bar (Figure 3). Again, the consumption of **Ru-imine** occurs in a first-order manner after a brief acceleration period. The k_obs_ value is constant with varying hydrogen pressure, indicating a partial order of zero under these conditions. The results in Figures 2 and 3 are consistent with an initial rate-determining step or sequence involving only **Ru-imine**; e.g. irreversible, rate-determining dissociation of PCy_3_, followed by incorporation of H_2_ after the rate-determining step.

### Variation of [PCy_3_]_0_

**Figure 4:**
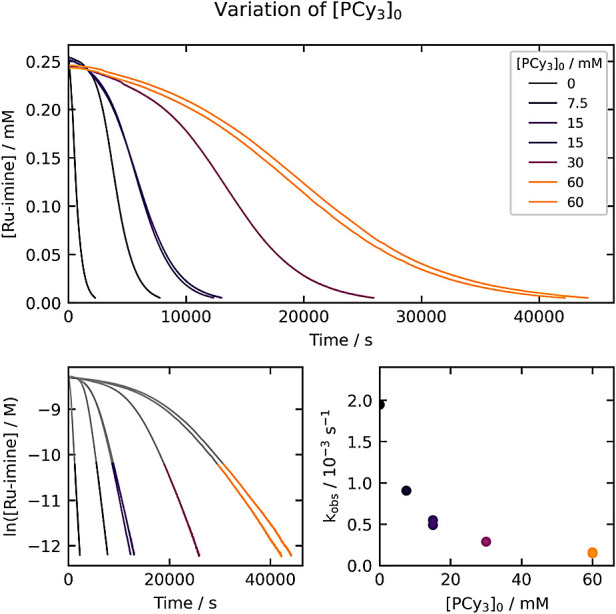
Kinetics of **Ru-imine** hydrogenation with varying [PCy_3_]_0_. The top plot shows the evolution of [**Ru-imine**] over time, and the bottom-left plot shows the evolution of ln[**Ru-imine**] over time. The bottom-right plot shows the dependence of k_obs_ on [PCy_3_]_0_. The k_obs_ values (top right) are derived from the period between 85% and 98% conversion, as highlighted in the bottom-left plots.

To probe whether dissociation of PCy_3_ might be a reversible initial step, we conducted experiments with added PCy_3_, where the initial PCy_3_ concentration ranged from 0 to 60 mM (Figure 4). Two important conclusions can be drawn from these experiments. First, the reaction is systematically slowed as [PCy_3_]_0_ is increased. This is consistent with reversible dissociation of PCy_3_ occurring before the rate-determining step. Second, the kinetic traces are sigmoidal in nature, with the acceleration period lasting several hours at the highest PCy_3_ concentration, too long to be explained by mass transfer of hydrogen into solution. This is suggestive of catalysis by the product **Ru-NH**, which is examined in the experiments described below.

In this series of experiments, we conducted two duplicate kinetic runs, with [PCy_3_]_0_ = 15 mM and 60 mM. As Figure 4 shows, the time courses for these runs are nearly identical, qualitatively indicating a high degree of reproducibility in these experiments. A more quantitative measure of the experimental error arises from the fit of the global data set to a microkinetic model, as described later.

### Variation of **[Ru-imine]_0_** at High [PCy_3_]_0_

**Figure 5:**
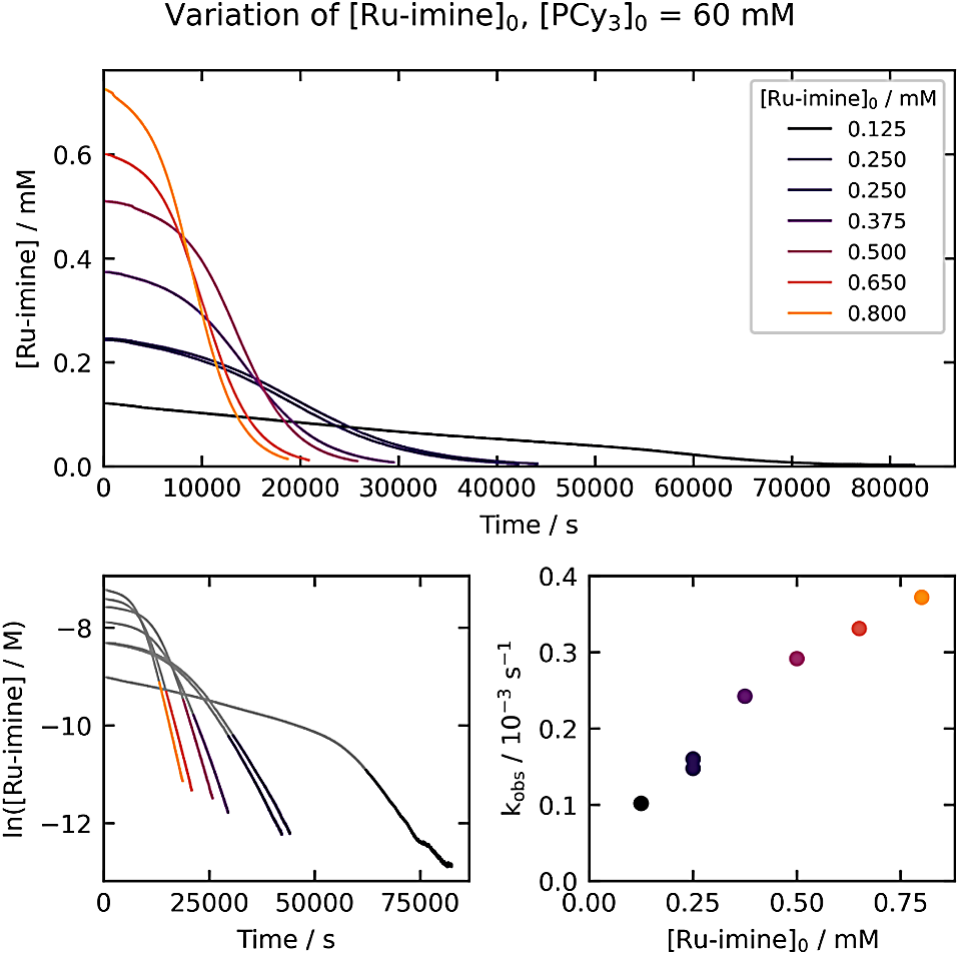
Kinetics of **Ru-imine** hydrogenation with varying [**Ru-imine**]_0_, with [PCy_3_]_0_ held constant at 60 mM. The top plot shows the evolution of [**Ru-imine**] over time, and the bottom-left plot shows the evolution of ln[**Ru-imine**] over time. The bottom-right plot shows the dependence of k_obs_ on [**Ru-imine**]_0_. The k_obs_ values (top right) are derived from the period between 85% and 98% conversion, as highlighted in the bottom-left plots.

To probe whether the sigmoidal kinetics at higher [PCy_3_]_0_ arise from autocatalytic behavior, we conducted experiments where [PCy_3_]_0_ was held constant at 60 mM and [**Ru-imine**]_0_ was varied from 0.125 mM to 0.800 mM (Figure 5). Qualitatively, the reactions are faster when the initial concentration of **Ru-imine** is higher, and all of the kinetic traces maintain the early acceleration periods. The pseudo-first-order k_obs_ values measured at the end of the reaction – from 85% to 98% conversion, when the concentration of the product **Ru-NH** is approximately constant – serve as a measure of the effect of [**Ru-NH**] on the reaction rate. As the bottom-right plot in Figure 5 shows, k_obs_ increases systematically as [**Ru-imine**]_0_ increases, consistent with product catalysis under these conditions.

### Variation of the Hydrogen Pressure at High [PCy_3_]_0_

**Figure 6:**
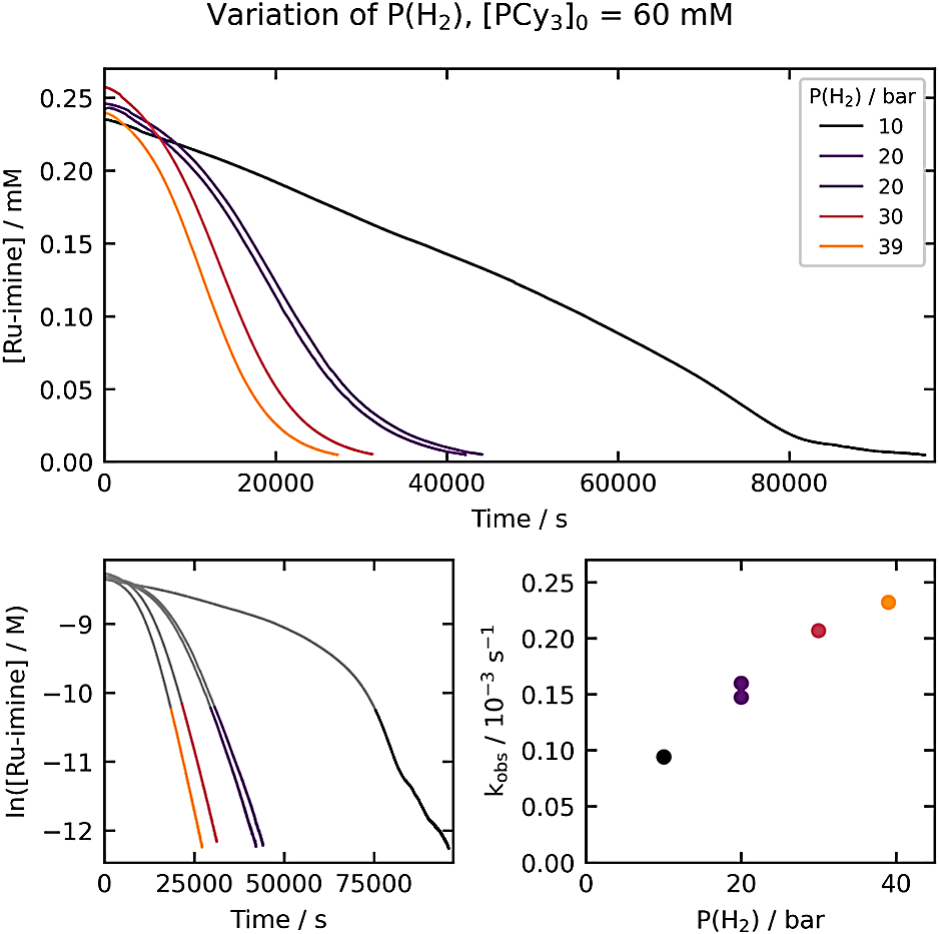
Kinetics of **Ru-imine** hydrogenation with varying hydrogen pressure, with [PCy_3_]_0_ held constant at 60 mM. The top plot shows the evolution of [**Ru-imine**] over time, and the bottom-left plot shows the evolution of ln[**Ru-imine**] over time. The bottom-right plot shows the dependence of k_obs_ on the hydrogen pressure. The k_obs_ values (top right) are derived from the period between 85% and 98% conversion, as highlighted in the bottom-left plots.

To determine whether hydrogen reacts prior to a potential autocatalytic rate-determining step at high [PCy_3_]_0_, we conducted experiments where [PCy_3_]_0_ was held constant at 60 mM and the hydrogen pressure was varied from 10 to 39 bar (Figure 6). Under these conditions, the reaction is faster at higher hydrogen pressures, in contrast to experiments with no added PCy_3_, where the hydrogen pressure had no effect on the reaction rate (see Figure 3). This is consistent with hydrogen incorporation prior to the rate-determining step when [PCy_3_]_0_ is high.

### Summary of the Kinetic Data

The kinetic experiments depicted in Figures 2–6 can be summarized as follows: 1) When no PCy_3_ is added, the hydrogenation of **Ru-imine** is first-order in [**Ru-imine**], with no effect on the reaction rate by the product **Ru-NH** or the hydrogen pressure; 2) addition of PCy_3_ systematically slows the reaction; and 3) when the initial concentration of PCy_3_ is high, the reaction shows a positive order in the product **Ru-NH** and the hydrogen pressure.

## Computational Mechanistic Analysis

Our search for energetically plausible mechanisms using DFT was guided by the kinetic data described above, which supports initial dissociation of PCy_3_ from **Ru-imine**, followed by two parallel pathways: 1) a direct pathway leading to **Ru-NH** through reaction with H_2_; and 2) a product-catalyzed pathway where **Ru-NH** transfers one or more equivalents of H_2_ to an intermediate formed after PCy_3_ dissociation from **Ru-imine**. In our DFT study using ORCA 6.0.0,^19^ we carried out geometry optimizations and frequency analyses with the r^2^SCAN-3c composite method,^20^ with single-point refinements at the ωB97X-D3^21^/def2-QZVPPD^22^ level. All calculations employed the SMD continuum solvation model for toluene.^23^ All structures were first optimized as closed-shell singlets, and this is the preferred electronic state for all species except for **a**, which optimizes as an open-shell singlet. We also assessed the possibility of competing triplet ground states for all structures. In most cases the singlet state was preferred as expected for second-row transition-metal complexes, but the singlet and triplet electronic states for **a** are close in energy, indicating potential multireference character. These comparisons are described in detail in the Supporting Information; all energies reported below represent the singlet electronic state.

### Direct Hydrogenation of **Ru-imine**

**Figure 7:**
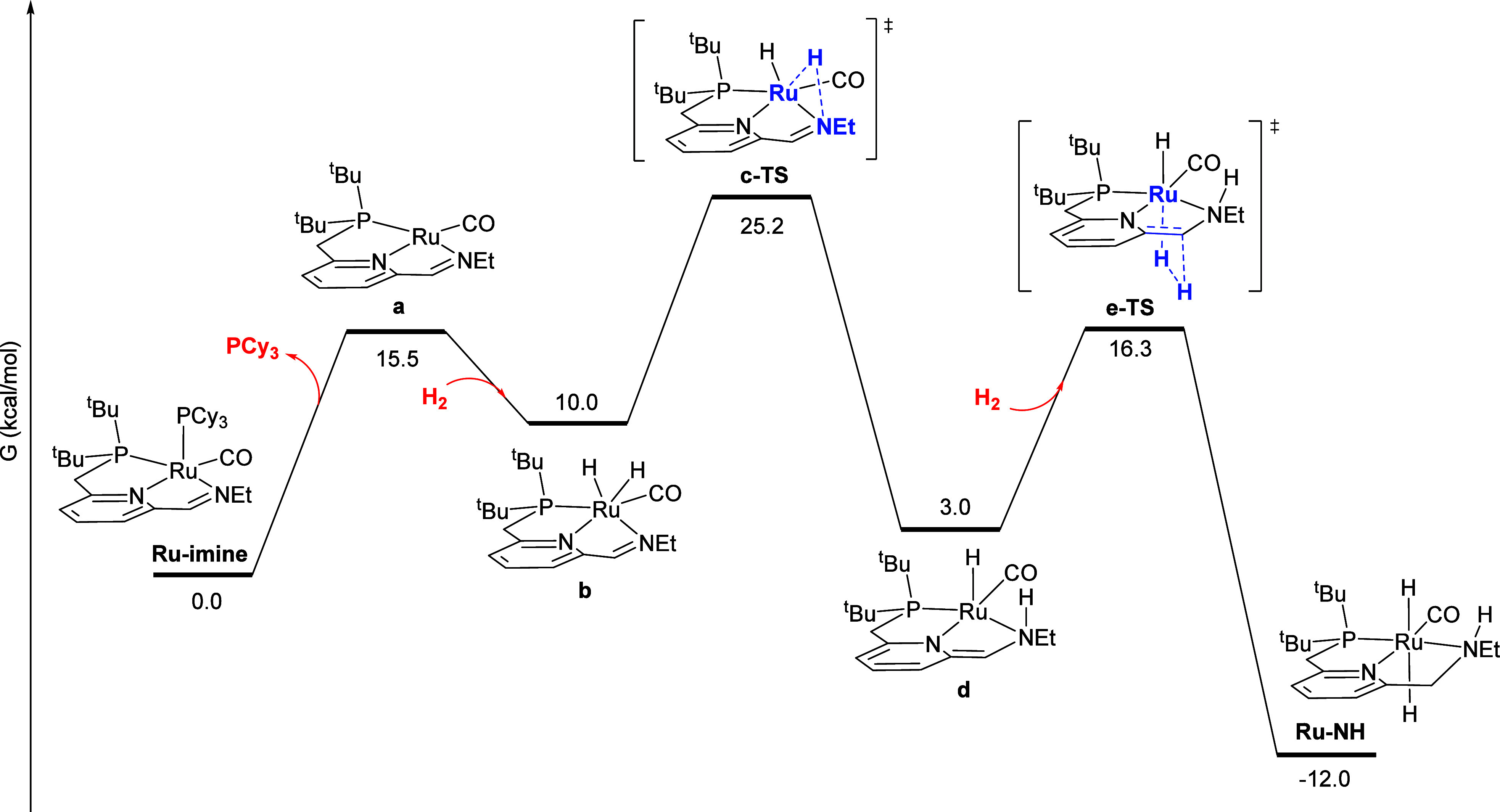
Minimum-energy pathway for the direct double hydrogenation of **Ru-imine** to give **Ru-NH**. For all DFT calculations, free energies are reported at the experimental reaction temperature of 298.15 K, relative to the reactant **Ru-imine**. For transition states, the atoms principally involved in forming and cleaving bonds are highlighted in blue and bold.

Figure 7 shows the minimum-energy pathway for the direct reaction of two equivalents of H_2_ with **Ru-imine** to give **Ru-NH** and PCy_3_. First, dissociation of PCy_3_ from **Ru-imine** gives the highly reactive four-coordinate Ru(0) complex **a**. We were not able to locate a transition state for the cleavage of the Ru-P bond; a relaxed scan (Figure S4) indicates that this dissociation proceeds without barrier on the electronic potential energy surface. Intermediate **a** then undergoes oxidative addition with H_2_ to give the ruthenium(II) intermediate **b**, where the pincer ligand distorts to a pseudofac coordination geometry and the nascent hydride ligands are mutually cis. In **b**, the cleavage of the H-H bond is essentially complete, as the H-H distance is 1.87 Å and Ru-H distances are each 1.61 Å. This oxidative addition also proceeds without barrier on the electronic potential energy surface, as confirmed by a relaxed scan (Figure S5). Then, the ruthenium(II)-dihydride **b** converts to **d** through **c-TS**, where a hydride ligand migrates from the ruthenium center to the imine nitrogen. In the conversion of **b** to **d**, ruthenium maintains its formal oxidation state of + 2, while the pyridine nitrogen becomes formally anionic and the pyridine ring is dearomatized. We note that **d** is structurally analogous to Milstein’s catalyst **Ru-dearom** (Scheme 1), except that the CH_2_ linker adjacent to nitrogen (rather than phosphorus) is deprotonated, and one N-Et group is replaced by N-H. Intermediate **d** then reacts with a second equivalent of H_2_ to give **Ru-NH**, through metal–ligand-cooperative addition of H_2_ to the Ru center and carbon linker.^24^

In addition to the minimum-energy pathway described above, we located a pathway for the metal–ligand-cooperative addition of H_2_ to the Ru center and imine carbon linker of **a** (Figure S6), and found an implausibly high free-energy barrier of 55.9 kcal/mol. We also identified a pathway where a ruthenium-bound hydride in **b** migrates to the imine carbon instead of the nitrogen (Figure S7). In this pathway, the ruthenium and carbon centers are too far apart for a low-barrier direct hydride migration. Instead, the imine C = N double bond first forms a π-complex with Ru, followed by hydride transfer from Ru to C. This sequence also proceeds with a higher barrier of 28.8 kcal/mol, compared to the minimum-energy pathway shown in Figure 7.

### Autocatalytic Hydrogenation of **Ru-imine**

**Figure 8:**
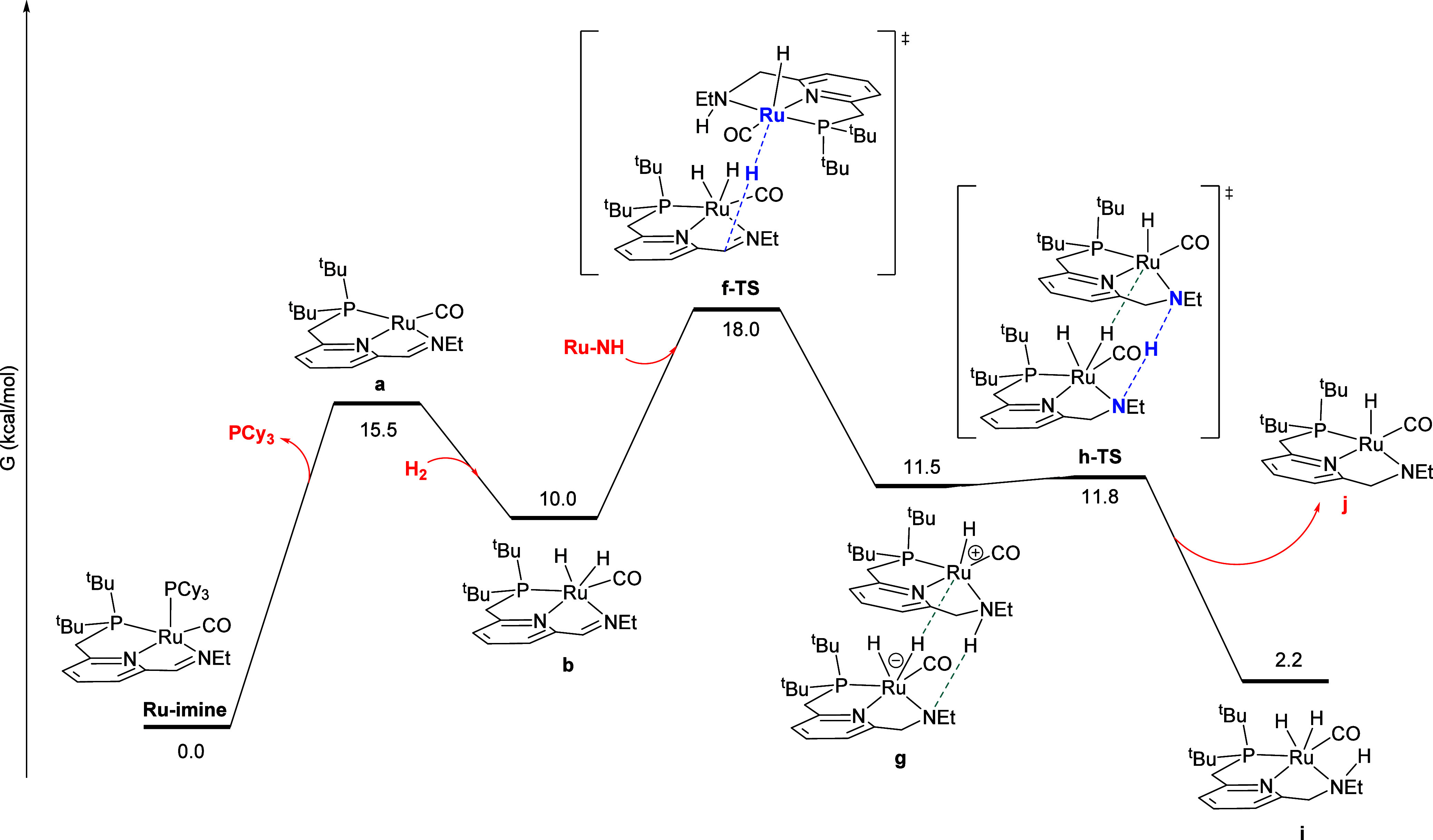
Minimum-energy pathway for the **Ru-NH**-catalyzed conversion of **Ru-imine** to intermediate **i**.

Figure 8 shows the first half of a sequence where the product **Ru-NH** catalyzes the hydrogenation of **Ru-imine**, by transferring an equivalent of H_2_ to the C = N double bond in **b**. As in the direct pathway above, this sequence begins with PCy_3_ dissociation to give **a** followed by barrierless oxidative addition of H_2_ to give **b**. Then, a molecule of **Ru-NH** transfers a hydride to the imine carbon of **b** to give the ion-pair intermediate **g** through **f-TS**, with a low barrier of 18.0 kcal/mol. Intermediate **g** then undergoes a nearly barrierless proton transfer through **h-TS**. The net result is the conversion of **b** to **i** (through hydrogenation of the C = N double bond) and the conversion of **Ru-NH** to **j** (through Noyori-type release of hydrogen from the RuH/NH moiety).

As described in more detail in the Supporting Information, we identified several alternatives to the minimum-energy pathway shown in Figure 8, all of which proceed with a higher barrier. First, we considered the possibility that **Ru-NH** transfers hydrogen to the C = N bond in intermediate **a** instead of intermediate **b**. Two diastereomeric versions of this pathway, shown in Figures S8 and S9, proceed with higher barriers of 25.1 and 24.4 kcal/mol, respectively. The reaction of **Ru-NH** with **a** instead of **c** would also be inconsistent with the kinetic data, as this pathway would be expected to be zero-order in hydrogen, while the experimental data (Figure 6) indicate that the product-catalyzed pathway has a positive order in hydrogen. Then, we considered a pathway where **Ru-NH** transfers a hydride to the imine nitrogen rather than the imine carbon, which results in a dearomatized pincer ligand (Figure S10). This pathway proceeded with a much higher barrier of 53.0 kcal/mol. Next, we considered pathways where ion-pair intermediates analogous to **g** (Figure 8) transfer a proton to Ru instead of N. These alternative pathways had higher barriers of 27.1 (Figure S11) and 27.8 kcal/mol (Figure S12). Last, we identified three diastereomeric versions for the hydride transfer from **Ru-NH** to **b** to give ion-pair intermediate **g**, with higher barriers of 22.5, 37.4, and 41.9 kcal/mol (Figures S13–S15).

**Figure 9:**
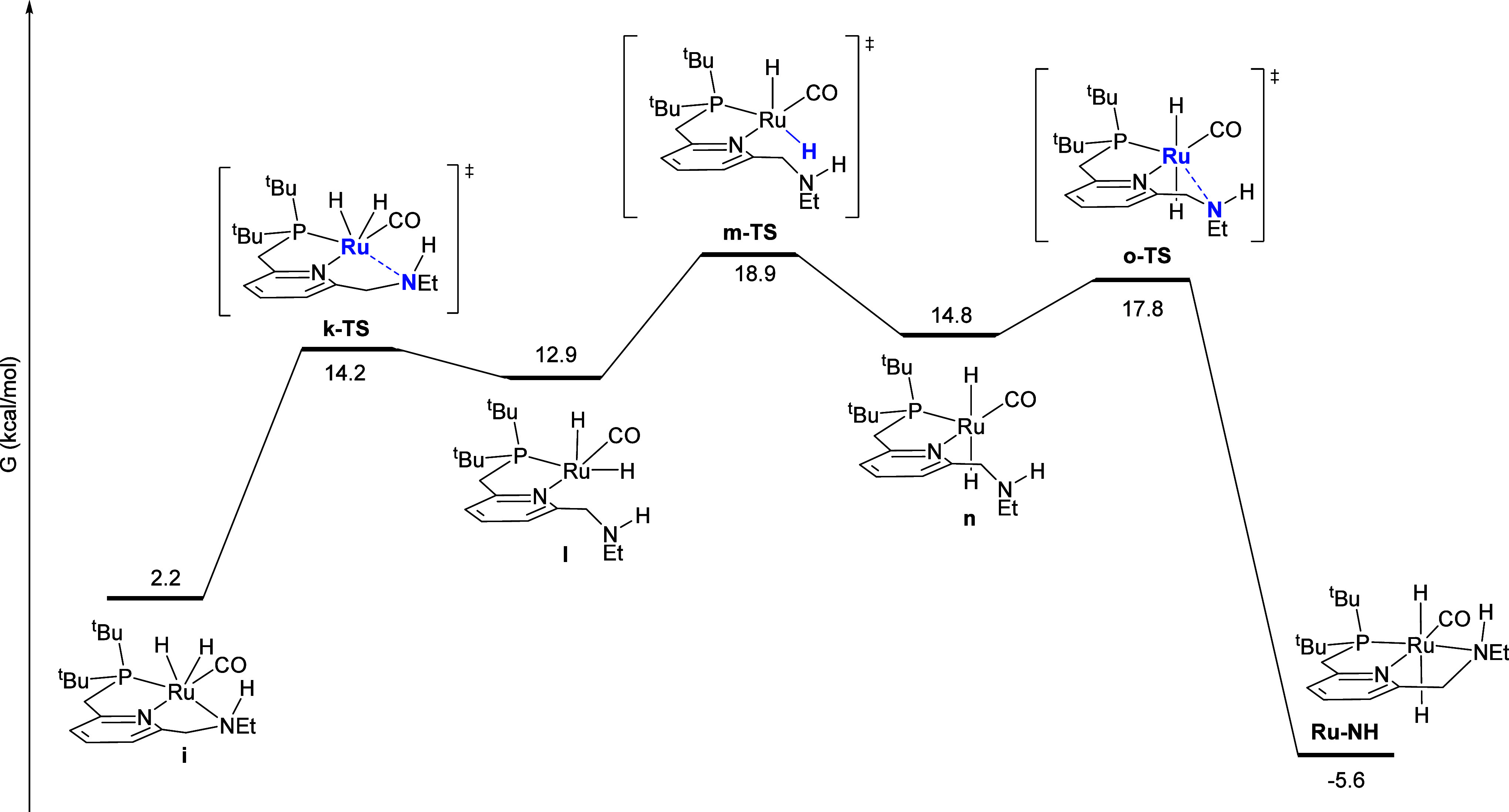
Minimum-energy pathway for the **isomerization** of intermediate **i** to the product **Ru-NH**.

Following the **Ru-NH**-mediated hydrogenation of the C = N bond in **b** to give the cis-dihydrido intermediate **i** (Figure 8), **i** isomerizes to the product **Ru-NH** by the minimum-energy pathway shown in Figure 9. First, the amine arm in **i** dechelates through **k-TS**, giving **l**. Then, the hydride trans to phosphorus migrates through y-shaped transition state **m-TS** to give **n**, where the hydrides are mutually trans. Lastly, the amine arm rechelates through **o-TS** to give the product **Ru-NH.**

**Figure 10:**
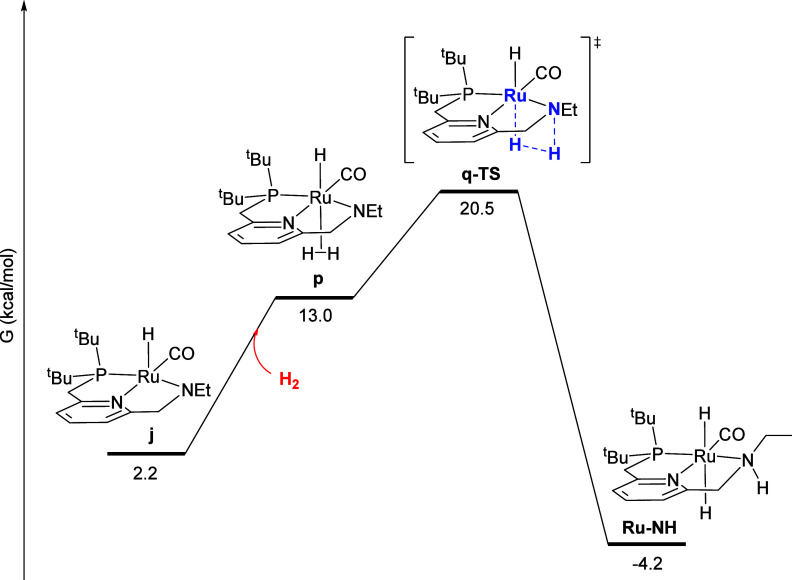
Minimum-energy pathway for the hydrogenation of intermediate **j** to regenerate the product **Ru-NH**.

To complete the autocatalytic reaction network, intermediate **j** – produced when **Ru-NH** transfers an equivalent of H_2_ to intermediate **b** – must be rehydrogenated. Figure 10 shows the well-precedented Noyori-type mechanism for this rehydrogenation, analogous to the manner in which **Ru-NH** acts as a catalyst for ester hydrogenation.^12^ First, H_2_ coordinates to the vacant site of **j** giving the H_2_ σ-complex **p**, with a H-H distance of 0.80 Å. Finally, hydrogen is cleaved through **q-TS**, giving the product **Ru-NH**.

## Kinetic Modeling with the Reaction Network Identified through DFT

**Scheme 4:**
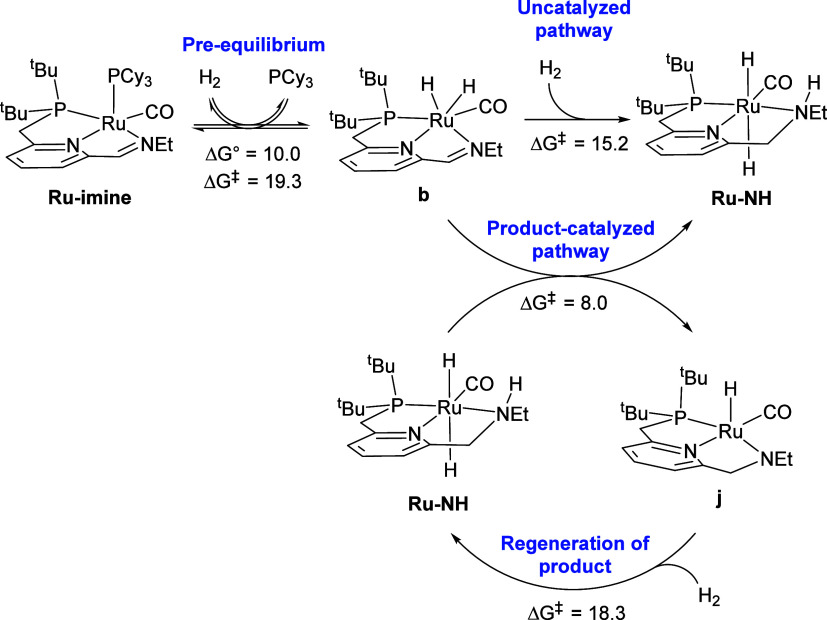
Kinetically Relevant Reaction Network for Product-Catalyzed Hydrogenation of **Ru-imine**

The reaction sequences in Figures 7–10 can be summarized by the kinetically relevant reaction network shown in Scheme 4. First, the reactant **Ru-imine** converts to **b**, releasing PCy_3_ and incorporating one molecule of H_2_, with a free-energy barrier of 19.3 kcal/mol, and a free-energy change of 10.0 kcal/mol. According to the kinetic data, this step is irreversible and rate-determining at low [PCy_3_]_0_ (Figures 2 and 3), but becomes a reversible pre-equilibrium at higher [PCy_3_]_0_ (Figure 4). Then, intermediate **b** converts to the product **Ru-NH** through two pathways, one uncatalyzed with a barrier from **b** of 15.2 kcal/mol (Figure 7), and the other product-catalyzed with a barrier from **b** of 8.0 kcal/mol, which also converts a molecule of **Ru-NH** to **j** (Figures 8 and 9). The kinetic data are consistent with catalysis by the product **Ru-NH** (Figure 5) and incorporation of hydrogen prior to the rate-determining, product-catalyzed step (Figure 6). The dehydrogenated intermediate **j** then reacts with hydrogen to regenerate the product **Ru-NH** with a free-energy barrier from **j** of 18.3 kcal/mol (Figure 10).

As a test of the validity of the reaction mechanism identified through DFT, we constructed a complete microkinetic model using the free program COPASI,^25^ including the forward and reverse reactions for every step shown in Figures 7–10. Because transition states were not located for the first two steps in Figures 7 and 8, or for the first step in Figure 10, the barriers for these bimolecular reactions were estimated following the suggestion of Harvey et al.,^26^ assuming that the barrierless association reaction is diffusion-controlled. In toluene at 298.15 K, a diffusion-controlled reaction proceeds with a rate constant of 1.12 × 10^10^ M^–1^·s^–1^, which corresponds to a free-energy barrier of 3.7 kcal/mol. Further details describing the microkinetic model are included in the Supporting Information. We then attempted to reproduce the kinetic data in Figures 2–6 by allowing the free energies of key rate-determining species to vary, with the goal of minimizing the number of freely varying parameters (free energies). By adjusting the free energies of intermediate **a** and transition states **c-TS** and **f-TS**, an excellent fit to the kinetic data was obtained (see below). Notably, the sequences in Figures 9 and 10 are not rate-determining; small adjustments of the free energies of **m-TS** (Figure 9) and **q-TS** (Figure 10) have no effect on the overall rate of reaction. Because the standard-state free energies of **m-TS** (Figure 9) and **q-TS** (Figure 10) are higher than that of **f-TS** (Figure 8), it may be counterintuitive that **f-TS** is rate-determining while the others are not. This apparent contradiction arises from the choice to reference all free energies against the overall reactants: the free energy of **m-TS**, accounting for mass balance, includes the unstable dehydrogenated intermediate **j**, which can rapidly convert back to **Ru-NH** through the separate low-barrier sequence in Figure 10. Analogously, the free energy of **q-TS** includes the unstable intermediate **i**, which also rapidly converts to **Ru-NH** by the pathway in Figure 9. Our microkinetic model does not reproduce the 3–5-min acceleration period observable in the fastest reactions with no added PCy_3_, which may arise from hydrogen mass-transfer into solution. As such, data from the first 300 s of each experiment was excluded from the fit. This exclusion has a very small effect on the adjusted free energies, as demonstrated in Figure S16.

**Figure 11:**
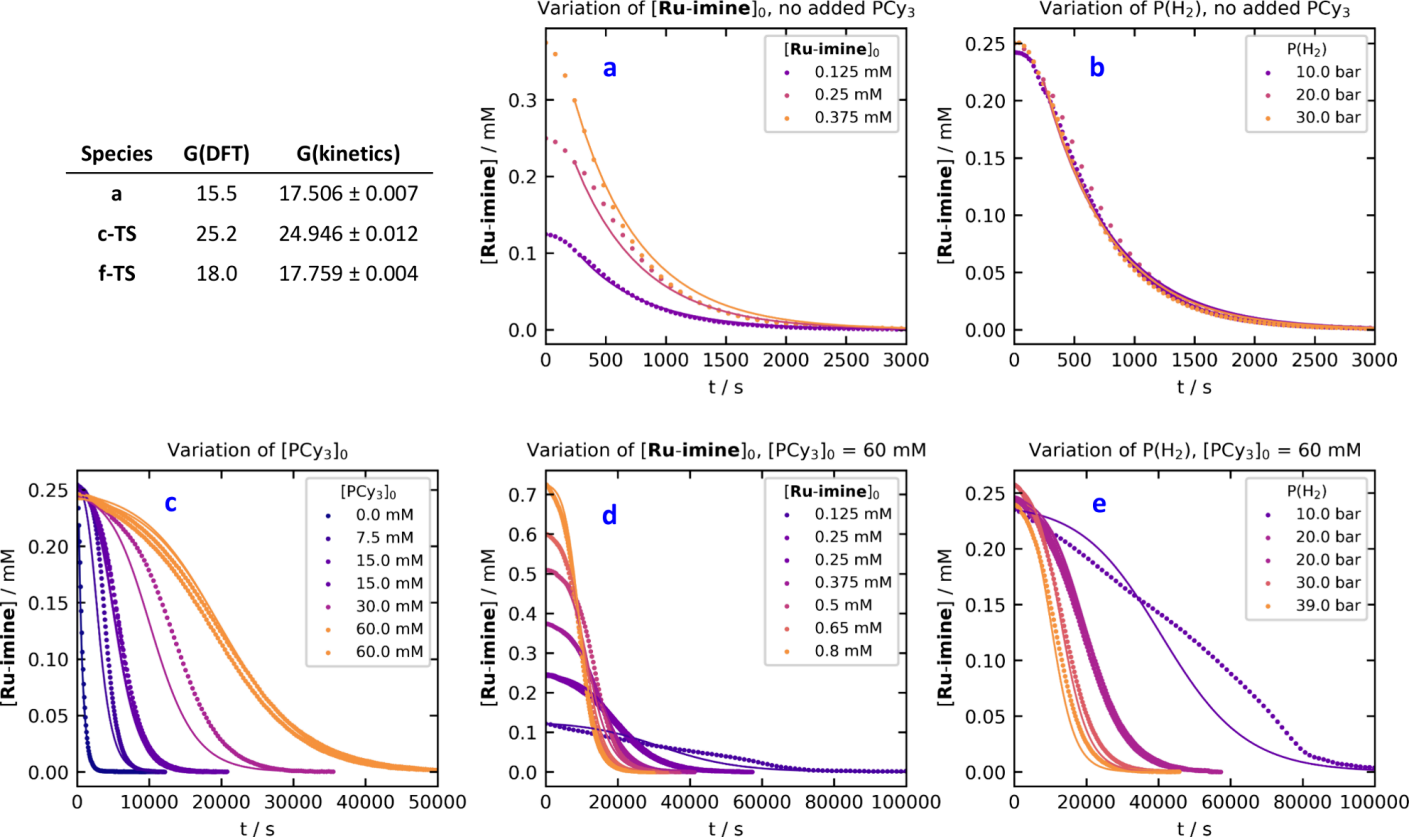
Comparison of the experimental kinetic data (points) and the time courses calculated with the optimized microkinetic model, after adjusting the free energies of **a**, **c-TS**, and **f-TS** to obtain the best fit. (a) Experiments where [**Ru-imine**]_0_ was varied with no added PCy_3_. (b) Experiments where the hydrogen pressure was varied with no added PCy_3_. (c) Experiments where [PCy_3_]_0_ was varied. (d) Experiments where [**Ru-imine**]_0_ was varied with 60 mM added PCy_3_. (e) Experiments where the hydrogen pressure was varied with 60 mM added PCy_3_.

Figure 11 shows a comparison of the kinetic data (points) against the time courses predicted using the microkinetic model (lines), after adjusting the free energies of **a**, **c-TS**, and **f-TS** to achieve the best fit. Details of the microkinetic model, including the data and model files used to conduct the analysis in COPASI, are included as Supporting Information. All three adjusted free energies are reasonably close to their values calculated by DFT. Since the free-energy barriers for the formation of **a** from **Ru-imine** and the reaction of **a** with H_2_ to form **b** (Figure 8) were estimated assuming a diffusion-controlled reaction, they were each set to be 3.7 kcal/mol higher than the free energy of **a**, and were dynamically adjusted to maintain this energy barrier as the free energy of **a** was adjusted.

Two repeated experiments shown in Figure 4 with [PCy_3_]_0_ = 15 mM and 60 mM qualitatively demonstrate the reproducibility of individual kinetic experiments. With a global kinetic model in hand, it is possible to estimate the overall experimental error through the uncertainties in the fitted free-energy values. These uncertainties, ranging from 0.004 for **f-TS** to 0.012 kcal/mol for **c-TS**, represent errors of 0.7% to 2.0% in the corresponding rate constants.

In addition to achieving a good overall fit to the kinetic data, the microkinetic model qualitatively reproduces the key trends observed in the kinetic experiments: 1) at low [PCy_3_]_0_, the reaction is pseudo-first-order in [**Ru-imine**], with no effect of [**Ru-imine**]_0_ or the hydrogen pressure; 2) as [PCy_3_]_0_ is increased, the reaction systematically slows and sigmoidal time courses are observed; 3) at high [PCy_3_]_0_, the reaction rate increases with increasing [**Ru-imine**]_0_ and hydrogen pressure. We note that although the sigmoidal shape of the kinetic traces is reproduced well in almost all cases, in the slowest reactions (Figure 11d, [**Ru-imine**]_0_ = 0.125 mM and Figure 11e, P(H_2_) = 10 bar), the initial consumption of **Ru-imine** is faster than the model predicts. While we are not certain of the origin of this deviation, we speculate that it might be due to very slow decomposition of **Ru-imine** through reaction with a trace contaminant, such as O_2_, which is most apparent when the hydrogenation of **Ru-imine** is slow.

## Conclusions

In summary, we have completed a detailed kinetic and computational study of the conversion of **Ru-imine**, the most active known additive-free precatalyst for ester hydrogenation,^11^ to its active form^12^**Ru-NH**. The data support an autocatalytic mechanism, where the product **Ru-NH** catalyzes the hydrogenation of the reactant **Ru-imine**, resulting in sigmoidal kinetic behavior. A microkinetic model based on the minimum-energy pathway identified through DFT is consistent with the kinetic data, providing support for the proposed mechanism. Because **Ru-imine** represents a PCy_3_-trapped form of the product of ethane release by Milstein’s catalyst **Ru-dearom**, the hydrogenative activation examined in this article is potentially relevant to related hydrogenation and dehydrogenation reactions catalyzed by **Ru-dearom**.^5−8,27^

## Methods

### General Methods

Toluene was purchased in anhydrous form from EMD-Millipore and was deoxygenated by sparging with argon before bringing into the glovebox. Tricyclohexylphosphine was recrystallized by layering a solution in toluene with acetonitrile and dried before use. Hydrogen gas was purchased from Airgas at the Ultrahigh Purity level. **Ru-imine** was synthesized as described previously.^11^

### Apparatus for Kinetic Experiments

Kinetic experiments were conducted in a stainless-steel pressure reactor, custom-built by Parr Instruments. The reactor was based on the Series 4790 Micro Non-Stirred Pressure Vessel, and included fittings at the top for hydrogen filling and venting, a safety rupture disk, an analog pressure gauge, and a temperature probe. Two ports were installed near the base, with fused silica windows and threads to accommodate SMA-905 fiber optic adapters. A stainless-steel cuvette holder for 1-cm square-based cuvettes was customized to fit inside the reactor. A schematic of the reactor is shown in Figure S2. For temperature control, the reactor was wrapped tightly with heating tape (Briskheat part no. BIH051040L), which, along with the reactor’s thermocouple, was connected to an Omega Platinum Series Universal Benchtop Digital Controller, part no. CS8DPT. To maintain a constant reactor temperature of 25 °C in a room varying from 19 to 22 °C, it was essential to continually fan the reactor using a simple desk fan. UV–visible radiation was provided with an Ocean Insight DH-2000-BAL Light Source, and detection was accomplished using a FLAME-S-UV–vis spectrometer from Ocean Insight. The light source and detector were connected to the reactor using 600 μm Premium Fiber Optic Cables, Ocean Insight part no. QP600–025-UV. A photo of the complete, assembled apparatus is shown in Figure S3.

### Experimental Procedure for Kinetic Experiments

As the reactant **Ru-imine** is extremely oxygen-sensitive, it was necessarily to rigorously exclude air from the reactor in kinetics experiments. The experimental apparatus, including light source, detector, stir plate, temperature controller, and fan, was assembled in a fume hood. The deuterium and tungsten lamps were turned on prior to assembling the reactor. The pressure reactor, along with the heating tape, was disconnected and brought into an argon-filled glovebox. Inside the glovebox, a reaction solution was prepared with the appropriate starting concentrations of **Ru-imine** and PCy_3_, which was transferred to an oven-dried 1-cm square-base quartz cuvette, along with a stir bar. The cuvette was inserted into the reactor. The reactor was then sealed, removed from the glovebox, and placed onto the stir plate, which was set to 500 rpm. The fiber optic cables were connected, and the heating tape and temperature probe were connected to the temperature controller. The fan was set to low speed. The temperature was allowed to stabilize at 25 °C, which typically required approximately 20 min. The hydrogen pressure line was purged for two minutes, then connected to the reactor under a gentle hydrogen flow. After pressurizing the line as appropriate, the reactor’s inlet needle valve was opened to pressurize the reactor. At the same time, UV–vis data collection was initiated. The raw UV–vis intensity spectrum was collected every 10 s over the course of the reaction. The intensity at 700 nm was used to calculate [**Ru-imine**] as a function of time, as described in the Supporting Information.

### Computational Methods

Density functional theory calculations were performed using the ORCA computational chemistry package, version 6.0.0.^19^ The geometries and energies of all species were calculated using the r^2^SCAN-3c composite method, developed by Grimme and coco-workers.^20^ The solvation-corrected electronic energies were further refined using the ωB97X-D3 functional from Head-Gordon and co-workers,^21^ along with the def2-QZVPPD basis set.^22^ For improved convergence of geometries, the DEFGRID3 integration grid was used for all calculations, along with the TIGHTSCF keyword to achieve tight SCF convergence. Geometry optimization, frequency calculations, and single-point energy refinements were conducted within a polarizable continuum with radii and nonelectrostatic terms from Truhlar and co-workers’ SMD solvation model, and with dielectric constants chosen for toluene.^23^ Complete structures with no truncations were used in all cases. Frequency calculations ensured the absence of imaginary vibrational modes in intermediates and the presence of exactly one imaginary mode in transition states. Intrinsic reaction coordinate calculations were employed to verify that transition states led to the specified minima. Free-energy corrections were calculated at the experimental reaction temperature of 25 °C, or 298.15 K. Standard-state corrections were applied in order to adjust from 1 atm to 1 M for solution-phase free energies, amounting to 1.89 kcal/mol added to the free energy of each isolated molecule at 298.15 K.^28^ Although the standard state for molecular hydrogen is sometimes taken as the gas at 1 atm, we have used a 1 M standard state in toluene, for consistency in computing reaction kinetics from the calculated free energies. For species likely to be a rate-determining intermediate or transition state, a conformational analysis was conducted using the CREST program developed by Grimme and coco-workers.^29^ All energies reported in the paper are standard-state free energies at 298.15 K. A table of energies is provided in the Supporting Information, and geometries in Cartesian coordinates are included in a separate, compiled .XYZ file.

For the closed-shell structures in this work, initial DFT calculations employed the default restricted SCF method, but the possibility of open-shell singlet electronic states was assessed by conducting stability analyses on all structures. While most structures were correctly described as closed-shell singlets, compound **a** optimized as an open-shell singlet, and the free energy shown in Figures 7 and 8 reflects this electronic structure. We also reoptimized each structure in the triplet state. For most structures, the triplet is significantly higher in energy, but for **a**, the singlet and triplet states are close in energy, indicating possible multireference character. A detailed discussion of this study of electronic states is included in the Supporting Information.

## Supporting Information Available

Additional experimental and computational details (PDF)
Atomic coordinates for all computed molecular structures, compiled as one file for singlets, readable by the free program Mercury^30^ (XYZ)
Atomic coordinates for all computed molecular structures, compiled as one file for triplets, readable by the free program Mercury^30^ (XYZ)
COPASI models, kinetic data in the format directly readable by COPASI, and COPASI output files comparing experimental data with fitted data (ZIP)

## Supplementary Material

cs4c04475_si_001.pdf

cs4c04475_si_002.xyz

cs4c04475_si_003.xyz
