# Supplementary material for: Autocatalytic Activation of a Ruthenium-PNN-Pincer Hydrogenation Catalyst
Source: ACS Catal. 2024 Oct 23;14(21):16497–507. doi: 10.1021/acscatal.4c04475 (PMC11536344; doi:10.1021/acscatal.4c04475)
Supplement: Supplementary file 1 — cs4c04475_si_001.pdf [file cs4c04475_si_001.pdf]

Supporting Information for

# Autocatalytic Activation of a Ruthenium-PNN-Pincer Hydrogenation Catalyst

Jose Fernando Carbajal Perez, Fallyn L. Kirlin, Eamon F. Reynolds, Cole E. Altomare-Jarczyk, Benjamin T. Joseph, Jason M. Keith*, and Anthony R. Chianese*

Department of Chemistry, Colgate University, 13 Oak Drive, Hamilton, New York 13346, United States

Emails: J. M. Keith, jkeith@colgate.edu; A. R. Chianese, achianese@colgate.edu

**Table of Contents**

UV-Vis Standard Series for Ru-imine ..... 2
Images of the Apparatus for Kinetic Experiments ..... 3
Calculation of [Ru-imine] from UV-Vis Intensity Data ..... 5
Singlet Energies calculated by DFT ..... 6
Relaxed Scan for $PCy_3$ Dissociation from Ru-imine ..... 9
Relaxed Scan for Oxidative addition of $H_2$ to Ruthenium Complex a ..... 10
Alternative Pathway: Initial addition of $H_2$ across Ru and C ..... 11
Alternative Pathway: Transfer of hydrogen from Ru to the imine carbon ..... 11
Alternative Pathway: Product Ru-NH transfers hydrogen to intermediate a instead of c ..... 12
Alternative Pathway: Product Ru-NH transfers hydride to the imine nitrogen ..... 13
Alternative Pathway: Product Ru-NH protonates ruthenium instead of nitrogen ..... 14
Alternative Pathway: Diastereomeric versions of the minimum-energy pathway shown in Figure 8 ..... 15
Microkinetic Model ..... 16
Analysis of the spin multiplicities of species calculated by DFT ..... 18
References ..... 23

**UV-Vis Standard Series for Ru-imine**

To determine the molar absorptivity of **Ru-imine** and establish linearity of the absorbance response to concentration at higher [**Ru-imine**], we prepared a standard series in toluene under inert atmosphere and recorded UV-vis spectra. Figure S1 shows the complete recorded spectra, as well as a plot of the absorbance at 700 nm against [**Ru-imine**]. The best fit line gives a molar absorptivity of 1,747.8 $L \cdot mol^{-1} \cdot cm^{-1}$ at 700 nm, which was used to calculate [**Ru-imine**] in the kinetics experiments described below.

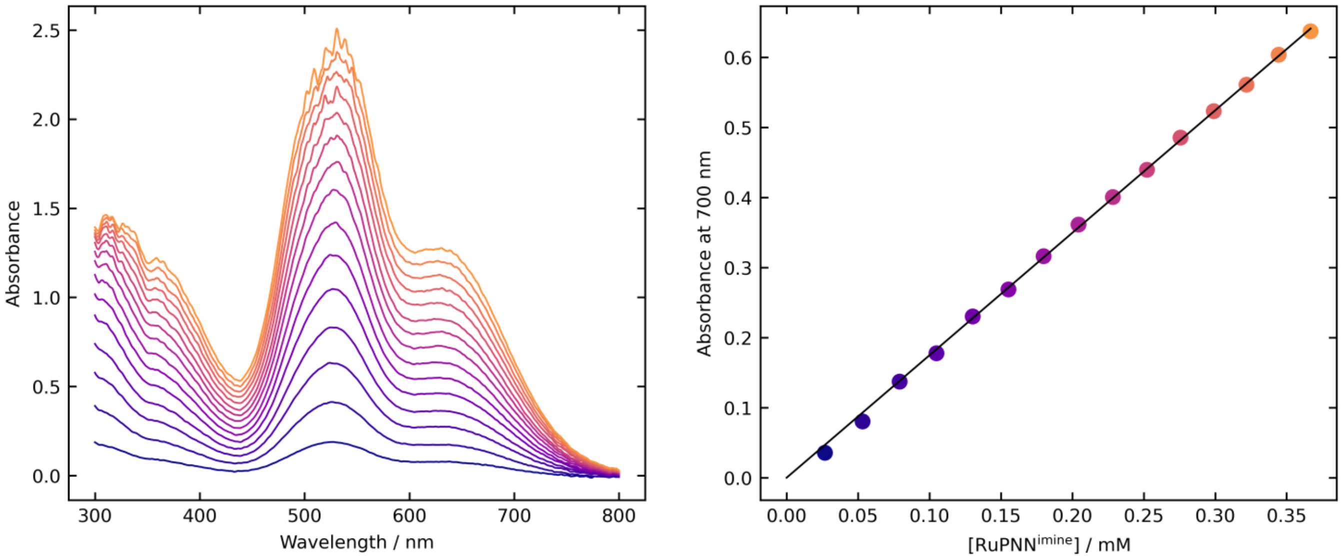


**Figure S1.** Standard series for Ru-imine. The left panel shows the UV-vis spectra obtained for each solution, and the right panel shows a plot of absorbance at 700 nm vs [Ru-imine].

**Images of the Apparatus for Kinetic Experiments**

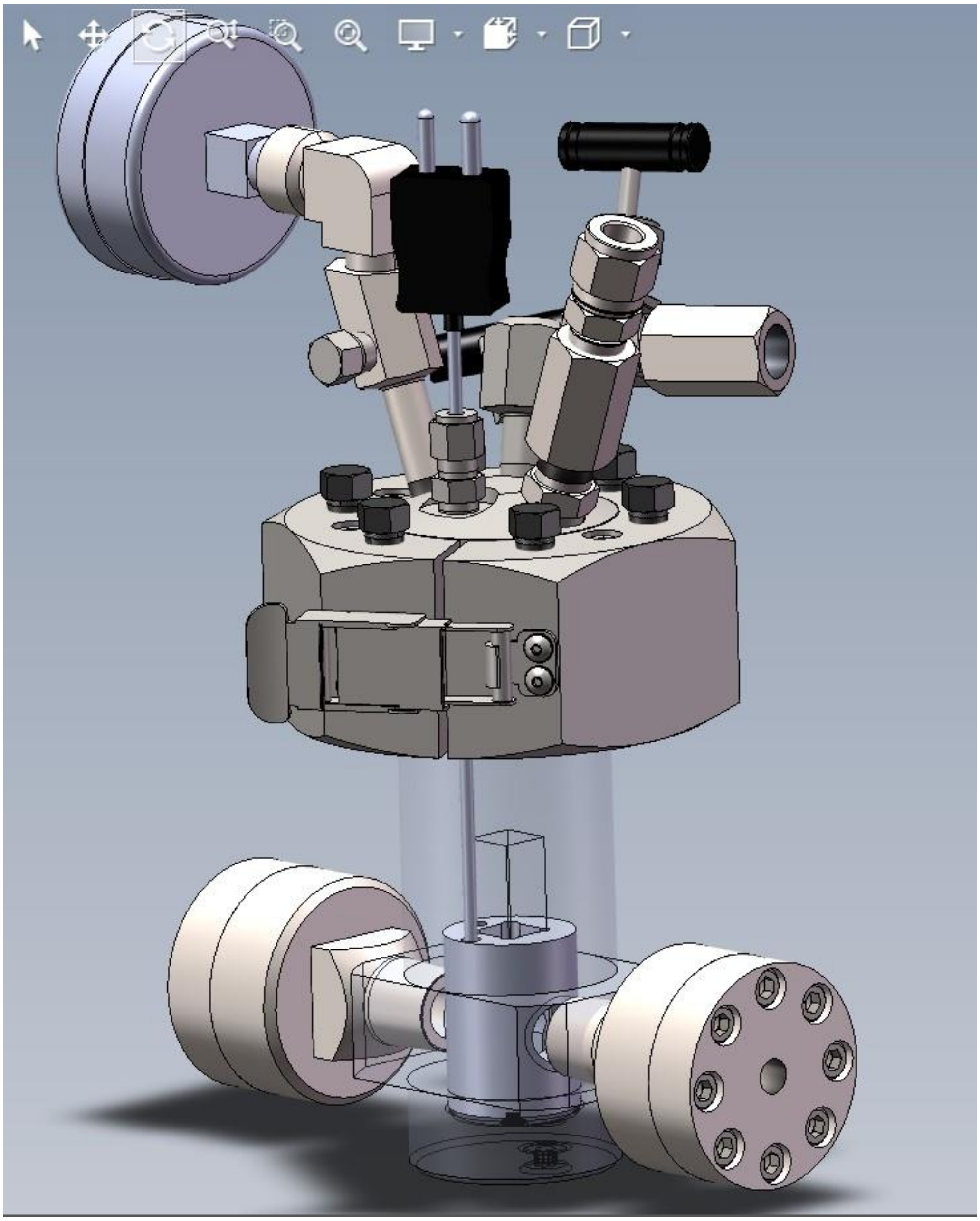

**Figure S2.** Schematic diagram of the custom pressure reactor designed for reaction monitoring by UV-vis.

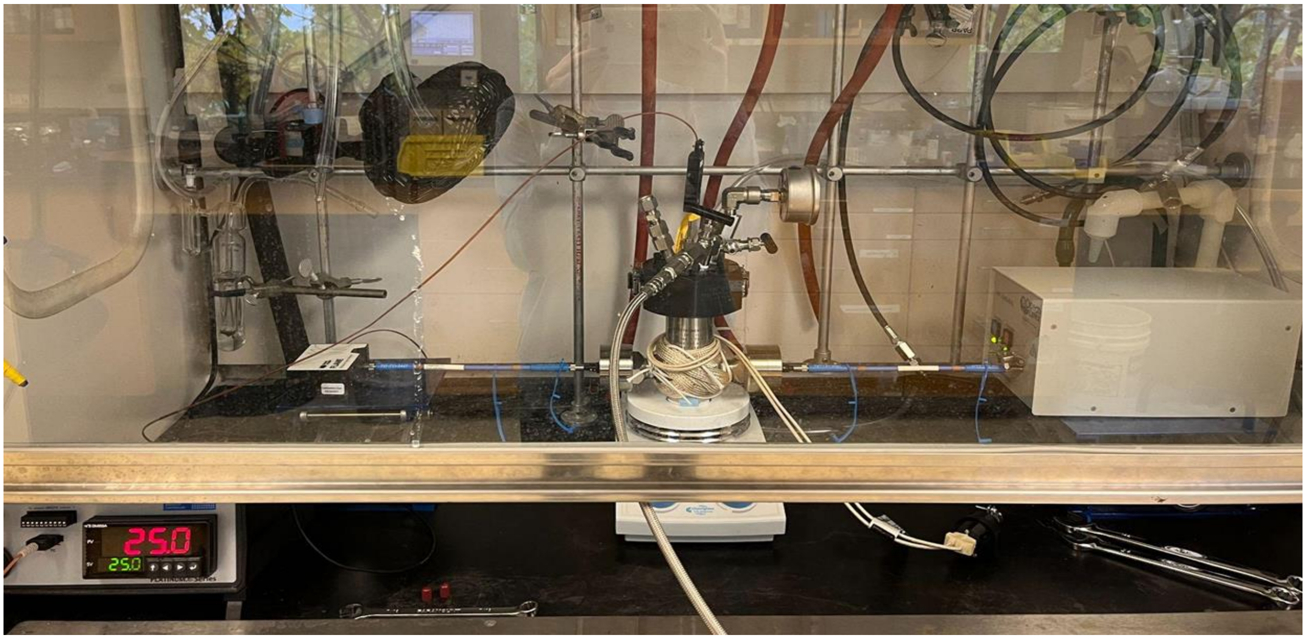


**Figure S3.** Photo of the complete, assembled reactor with a reaction in progress.

**Calculation of [Ru-imine] from UV-Vis Intensity Data**

Because the measured light intensity in each experiment is affected by variables including the precise position of the reactor, the fiber optic cables, and the position of the cuvette inside the reactor, it was not possible to collect accurate blank intensity spectra to calculate absorbance in the traditional manner. To calculate the absorbance at 700 nm and subsequently [**Ru-imine**] for each time point, we used its known molar absorptivity of 1,747.8 $L \cdot mol^{-1} \cdot cm^{-1}$, and we assumed that the blank intensity was equal to the maximum intensity measured over the course of the experiment. This calculation assumes that the product absorbance is negligible, which was validated by monitoring the reaction in a traditional UV-vis setup under 1 atmosphere of hydrogen (Figure 1 in the main text).

First, all measured intensities at all time points at 700 nm were corrected by subtracting the "dark" signal, which arises from electronic noise and can be measured by excluding all light from the detector:

$$\mathrm{I_t = I_{t-raw} - I_{dark}}$$

Next, absorbance at each time point was calculated, assuming that the blank intensity $I_0$ is equal to the maximum intensity measured over course of the experiment:

$$\mathrm{A_t} = -\log\left(\frac{\mathrm{I_t}}{\mathrm{I_0}}\right)$$

Finally, [**Ru-imine**] at each time point was calculated using the Beer-Lambert law:

$$[\textbf{Ru-imine}] = \frac{\mathrm{A_t}}{1747.8\ \mathrm{L \cdot mol^{-1} \cdot cm^{-1}} \times 1\ \mathrm{cm}}$$

The calculated concentration data for each experiment, reduced to include between 100 and 200 time points for each experiment, are included in the attached text files "copasi_input_trim_first_300_seconds.txt" and "copasi_input_all_data.txt", which are also formatted for direct use in kinetic modeling by the program COPASI.

**Singlet Energies calculated by DFT**

Table S1 below shows the energies calculated by DFT for all singlet structures reported in this paper. The column **E(opt)** represents the solvent-corrected electronic energy in hartrees, calculated at the end of geometry optimization with the $r^2$SCAN-3c composite method. **E(opt)** is not used in the calculation of the reported free energies, but is provided for completeness. The column **E(single point)** represents the solvent-corrected electronic energy in hartrees, calculated with the ωB97X-D3 functional and the def2-QZVPPD basis set. The column **G(corr)** represents the correction to the Gibbs free energy calculated at 298.15 K after geometry optimization using the $r^2$SCAN-3c composite method. The column **Imag.** shows the imaginary vibrational frequency calculated for transition states. The column **G(kcal)** represents the Gibbs free energy for each isolated species at 298.15 K in kcal/mol, calculated as the sum of **E(single point)** and **G(corr)**, and including the addition of 1.89 kcal/mol for each molecule to convert to a 1 M standard state. The column **Mass Balance** lists the small molecules included in the total free energy for the calculation of reaction pathways. The column **G(tot)** is the sum of standard-state free energies of the ruthenium complex in focus and any additional molecules included for mass balance, in kcal/mol. The column **G(rel)** is the total free energy in kcal/mol referenced against **Ru-imine**. A stability analysis showed that most species were correctly defined as closed-shell singlets. However, **a** optimizes as an open-shell singlet. The energy values for **a** in Table S1 represent the open-shell singlet structure.

## Table S1. Energies of singlet structures calculated by DFT.

| Small Molecules | E(opt) | E(single point) | G(corr) | Imag. | G(kcal) | Mass balance | G(tot) | G(rel) |
|---|---|---|---|---|---|---|---|---|
| hydrogen | -1.168938352 | -1.175355 | -0.001917 | | -736.8555673 | | | |
| $PCy_3$ | -1046.961231 | -1047.351990 | 0.434993 | | -656947.9448 | | | |
| **Direct Hydrogenation MEP (Fig. 7)** | | | | | | **Mass balance** | **G(tot)** | **G(rel)** |
| Ru-imine | -2371.330922 | -2372.082768 | 0.853231 | | -1487965.98 | 2 $H_2$ | -1489439.69 | **0.0** |
| a | -1324.312279 | -1324.678319 | 0.387531 | | -831002.49 | 2 $H_2$ + $PCy_3$ | -1489424.15 | **15.5** |
| b | -1325.510134 | -1325.880709 | 0.406796 | | -831744.92 | $H_2$ + $PCy_3$ | -1489429.72 | **10.0** |
| c-TS | -1325.47919 | -1325.852926 | 0.403204 | -1079.83 | -831729.74 | $H_2$ + $PCy_3$ | -1489414.54 | **25.2** |
| d | -1325.508667 | -1325.892744 | 0.407775 | | -831751.85 | $H_2$ + $PCy_3$ | -1489436.65 | **3.0** |
| e-TS | -1326.676011 | -1327.061176 | 0.423143 | -680.76 | -832475.41 | $PCy_3$ | -1489423.36 | **16.3** |
| Ru-NH | -1326.717257 | -1327.113499 | 0.430327 | | -832503.74 | $PCy_3$ | -1489451.68 | **-12.0** |
| **Autocatalytic MEP (Figs. 8 and 9)** | | | | | | | | |
| Ru-imine | -2371.330922 | -2372.082768 | 0.853231 | | -1487965.98 | 2 $H_2$ + Ru-NH | -2321943.43 | **0.0** |
| a | -1324.312279 | -1324.678319 | 0.387531 | | -831002.49 | 2 $H_2$ + Ru-NH + $PCy_3$ | -2321927.89 | **15.5** |
| b | -1325.510134 | -1325.880709 | 0.406796 | | -831744.92 | $H_2$ + Ru-NH + $PCy_3$ | -2321933.46 | **10.0** |
| f-TS | -2652.22929 | -2652.999733 | 0.858375 | -490.29 | -1664240.68 | $H_2$ + $PCy_3$ | -2321925.48 | **18.0** |
| g | -2652.238145 | -2653.017472 | 0.865877 | | -1664247.10 | $H_2$ + $PCy_3$ | -2321931.90 | **11.5** |
| h-TS | -2652.233693 | -2653.010927 | 0.859752 | -1050.9 | -1664246.84 | $H_2$ + $PCy_3$ | -2321931.64 | **11.8** |
| i | -1326.705508 | -1327.100857 | 0.430094 | | -832495.95 | $H_2$ + $PCy_3$ + j | -2321941.24 | **2.2** |
| k-TS | -1326.677541 | -1327.077285 | 0.425700 | -34.97 | -832483.92 | $H_2$ + $PCy_3$ + j | -2321929.20 | **14.2** |
| l | -1326.679891 | -1327.078964 | 0.425307 | | -832485.22 | $H_2$ + $PCy_3$ + j | -2321930.50 | **12.9** |
| m-TS | -1326.668634 | -1327.068519 | 0.424420 | -297.31 | -832479.22 | $H_2$ + $PCy_3$ + j | -2321924.50 | **18.9** |
| n | -1326.676472 | -1327.074985 | 0.424327 | | -832483.33 | $H_2$ + $PCy_3$ + j | -2321928.62 | **14.8** |
| o-TS | -1326.672096 | -1327.071375 | 0.425454 | -52.98 | -832480.36 | $H_2$ + $PCy_3$ + j | -2321925.65 | **17.8** |
| Ru-NH | -1326.717257 | -1327.113499 | 0.430327 | | -832503.74 | $H_2$ + $PCy_3$ + j | -2321949.02 | **-5.6** |
| **Rehydrogenation of j (Fig. 10)** | | | | | | | | |
| j | -1325.521075 | -1325.906121 | 0.407399 | | -831760.48 | $H_2$ + i + $PCy_3$ | -2321941.24 | **2.2** |
| p | -1326.684984 | -1327.079210 | 0.423472 | | -832486.52 | i + $PCy_3$ | -2321930.42 | **13.0** |
| q-TS | -1326.669778 | -1327.065154 | 0.421306 | -1107.85 | -832479.06 | i + $PCy_3$ | -2321922.96 | **20.5** |
| Ru-NH | -1326.717257 | -1327.113499 | 0.430327 | | -832503.74 | i + $PCy_3$ | -2321947.63 | **-4.2** |
| **Initial addition of $H_2$ across Ru and C (Fig. S6)** | | | | | | | | |
| a | -1324.312279 | -1324.678319 | 0.387531 | | -831002.49 | 2 $H_2$ + $PCy_3$ | -1489424.15 | **15.5** |
| r-TS | -1325.433477 | -1325.800676 | 0.399910 | -1351.06 | -831699.02 | $H_2$ + $PCy_3$ | -1489383.82 | **55.9** |
| j | -1325.521075 | -1325.906121 | 0.407399 | | -831760.48 | $H_2$ + $PCy_3$ | -1489445.28 | **-5.6** |
| **Transfer of hydrogen from Ru to the imine carbon (Fig. S7)** | | | | | | | | |
| b | -1325.510134 | -1325.880709 | 0.406796 | | -831744.92 | $H_2$ + $PCy_3$ | -1489429.72 | **10.0** |
| s | -1325.473233 | -1325.852383 | 0.404316 | | -831728.70 | $H_2$ + $PCy_3$ | -1489413.50 | **26.2** |
| t-TS | -1325.464645 | -1325.846525 | 0.402565 | -617.46 | -831726.12 | $H_2$ + $PCy_3$ | -1489410.92 | **28.8** |
| j | -1325.521075 | -1325.906121 | 0.407399 | | -831760.48 | $H_2$ + $PCy_3$ | -1489445.28 | **-5.6** |

**Table S1 continued. Energies calculated by DFT.**

| | E(opt) | E(single point) | G(corr) | Imag. | G(kcal) | Mass balance | G(tot) | G(rel) |
|---|---|---|---|---|---|---|---|---|
| **Product Ru-NH transfers hydrogen to intermediate a instead of b (Fig. S8)** | | | | | | | | |
| Ru-imine | -2371.330922 | -2372.082768 | 0.853231 | | -1487965.98 | 2 $H_2$ + Ru-NH | -2321943.43 | **0.0** |
| a | -1324.312279 | -1324.678319 | 0.387531 | | -831002.49 | 2 $H_2$ + Ru-NH + $PCy_3$ | -2321927.89 | **15.5** |
| u-TS | -2651.042524 | -2651.800382 | 0.842783 | -411.99 | -1663497.86 | 2 $H_2$ + $PCy_3$ | -2321919.52 | **23.9** |
| v | -2651.046312 | -2651.806564 | 0.844290 | | -1663500.79 | 2 $H_2$ + $PCy_3$ | -2321922.45 | **21.0** |
| w-TS | -2651.032262 | -2651.798923 | 0.843141 | -763.03 | -1663496.72 | 2 $H_2$ + $PCy_3$ | -2321918.37 | **25.1** |
| x | -1325.50744 | -1325.892958 | 0.411713 | | -831749.52 | 2 $H_2$ + $PCy_3$ + j | -2321931.66 | **11.8** |
| i | -1326.705508 | -1327.100857 | 0.430094 | | -832495.95 | $H_2$ + $PCy_3$ + j | -2321941.24 | **2.2** |
| **Product Ru-NH transfers hydrogen to intermediate a instead of b (Fig. S9)** | | | | | | | | |
| Ru-imine | -2371.330922 | -2372.082768 | 0.853231 | | -1487965.98 | 2 $H_2$ + Ru-NH | -2321943.43 | **0.0** |
| a | -1324.312279 | -1324.678319 | 0.387531 | | -831002.49 | 2 $H_2$ + Ru-NH + $PCy_3$ | -2321927.89 | **15.5** |
| u1-TS | -2651.040843 | -2651.799231 | 0.842371 | -377.24 | -1663497.40 | 2 $H_2$ + $PCy_3$ | -2321919.05 | **24.4** |
| v1 | -2651.048113 | -2651.807819 | 0.845119 | | -1663501.06 | 2 $H_2$ + $PCy_3$ | -2321922.72 | **20.7** |
| w1-TS | -2651.038621 | -2651.797161 | 0.840062 | -940.86 | -1663497.55 | 2 $H_2$ + $PCy_3$ | -2321919.20 | **24.2** |
| x | -1325.50744 | -1325.892958 | 0.411713 | | -831749.52 | 2 $H_2$ + $PCy_3$ + j | -2321931.66 | **11.8** |
| i | -1326.705508 | -1327.100857 | 0.430094 | | -832495.95 | $H_2$ + $PCy_3$ + j | -2321941.24 | **2.2** |
| **Product Ru-NH transfers hydrogen to imine nitrogen in b instead of imine carbon (Fig. S10)** | | | | | | | | |
| b | -1325.510134 | -1325.880709 | 0.406796 | | -831744.92 | $H_2$ + Ru-NH + $PCy_3$ | -2321933.46 | **10.0** |
| z-TS | -2652.18622 | -2652.946645 | 0.861109 | -1242.44 | -1664205.65 | $H_2$ + $PCy_3$ | -2321890.45 | **53.0** |
| aa | -2652.247044 | -2653.031445 | 0.864815 | | -1664256.54 | $H_2$ + $PCy_3$ | -2321941.34 | **2.1** |
| **Product Ru-NH protonates ruthenium instead of nitrogen (Fig. S11)** | | | | | | | | |
| v | -2651.046312 | -2651.806564 | 0.844290 | | -1663500.79 | 2 $H_2$ + $PCy_3$ | -2321922.45 | **21.0** |
| y-TS | -2651.036586 | -2651.793320 | 0.840866 | -672.48 | -1663494.63 | 2 $H_2$ + $PCy_3$ | -2321916.29 | **27.1** |
| j | -1325.521075 | -1325.906121 | 0.407399 | | -831760.48 | 2 $H_2$ + $PCy_3$ + j | -2321942.62 | **0.8** |
| **Product Ru-NH protonates ruthenium instead of nitrogen (Fig. S12)** | | | | | | | | |
| v1 | -2651.048113 | -2651.807819 | 0.845119 | | -1663501.06 | 2 $H_2$ + $PCy_3$ | -2321922.72 | **20.7** |
| y2-TS | -2651.03627 | -2651.792945 | 0.841573 | -553.01 | -1663493.95 | 2 $H_2$ + $PCy_3$ | -2321915.61 | **27.8** |
| j | -1325.521075 | -1325.906121 | 0.407399 | | -831760.48 | 2 $H_2$ + $PCy_3$ + j | -2321942.62 | **0.8** |
| **Diastereomeric version of the MEP shown in Figure 8 (Fig. S13)** | | | | | | | | |
| b | -1325.510134 | -1325.880709 | 0.406796 | | -831744.92 | $H_2$ + Ru-NH + $PCy_3$ | -2321933.46 | **10.0** |
| f2-TS | -2652.222041 | -2652.991996 | 0.857860 | -342.71 | -1664236.15 | $H_2$ + $PCy_3$ | -2321920.95 | **22.5** |
| g2 | -2652.250361 | -2653.030991 | 0.863171 | | -1664257.28 | $H_2$ + $PCy_3$ | -2321942.08 | **1.3** |
| **Diastereomeric version of the MEP shown in Figure 8 (Fig. S14)** | | | | | | | | |
| b | -1325.510134 | -1325.880709 | 0.406796 | | -831744.92 | $H_2$ + Ru-NH + $PCy_3$ | -2321933.46 | **10.0** |
| f3-TS | -2652.207164 | -2652.970849 | 0.860510 | -508.23 | -1664221.21 | $H_2$ + $PCy_3$ | -2321906.01 | **37.4** |
| g3 | -2652.221781 | -2652.997251 | 0.863186 | | -1664236.10 | $H_2$ + $PCy_3$ | -2321920.90 | **22.5** |
| **Diastereomeric version of the MEP shown in Figure 8 (Fig. S15)** | | | | | | | | |
| b | -1325.510134 | -1325.880709 | 0.406796 | | -831744.92 | $H_2$ + Ru-NH + $PCy_3$ | -2321933.46 | **10.0** |
| f4-TS | -2652.198175 | -2652.963734 | 0.860617 | -773.84 | -1664216.68 | $H_2$ + $PCy_3$ | -2321901.48 | **41.9** |
| g4 | -2652.236754 | -2653.020414 | 0.860747 | | -1664252.17 | $H_2$ + $PCy_3$ | -2321936.97 | **6.5** |

### Relaxed Scan for $PCy_3$ Dissociation from Ru-imine

Figure S4 shows the electronic energies for a relaxed scan increasing the Ru-P distance in **Ru-imine**. This scan results in a smooth dissociation with small discontinuities and no minimum, and indicates that dissociation of $PCy_3$ (Figures 7 and 8) is barrierless on the electronic PES.

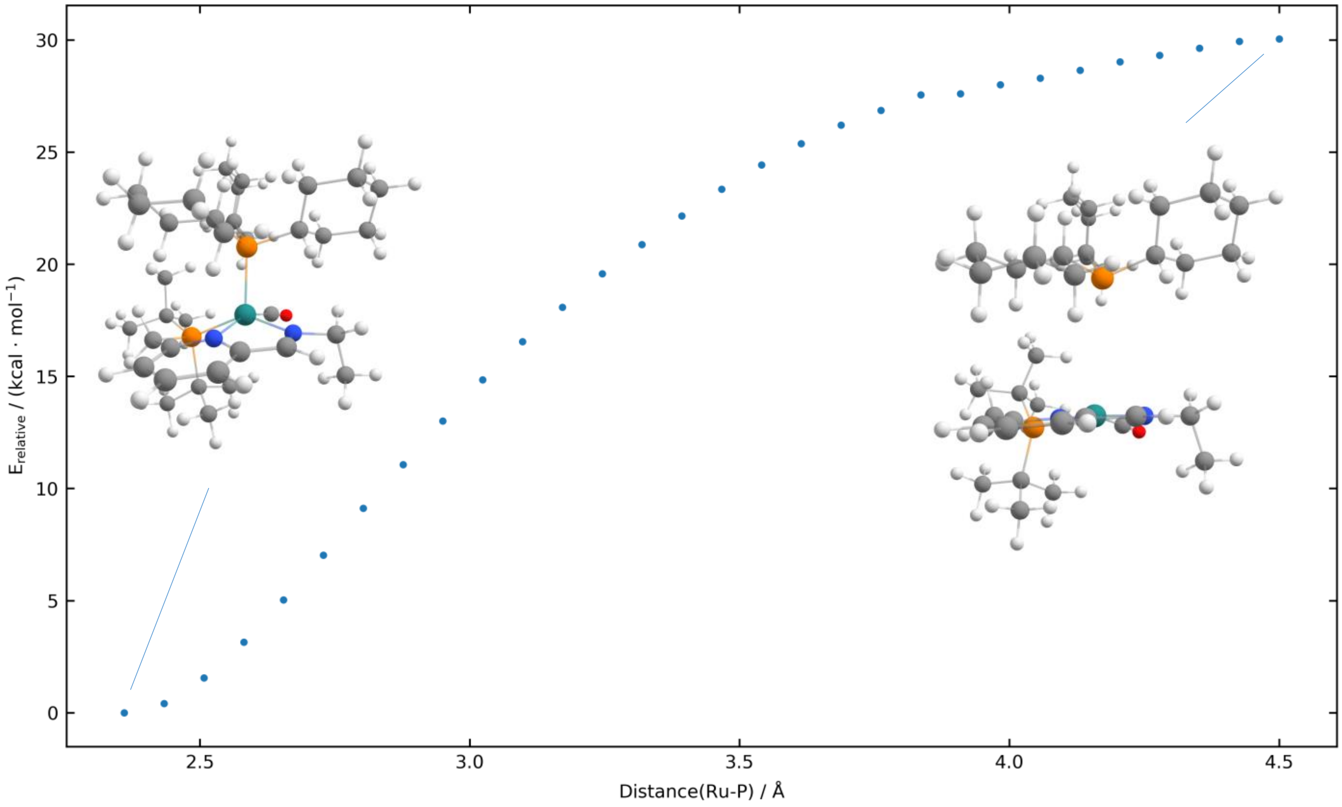


**Figure S4.** Relaxed scan for the dissociation of $PCy_3$ from **Ru-imine** to give **a**.

**Relaxed Scan for Oxidative addition of $H_2$ to Ruthenium Complex a**

Figure S5 shows the electronic energies for a relaxed scan where the H-H distance was decreased, starting from compound **b**. This scan results in a smooth reductive elimination of $H_2$ from **b**, with no minimum, and indicates that the reverse oxidative addition of $H_2$ to **a** (Figures 7 and 8) is barrierless on the electronic PES.

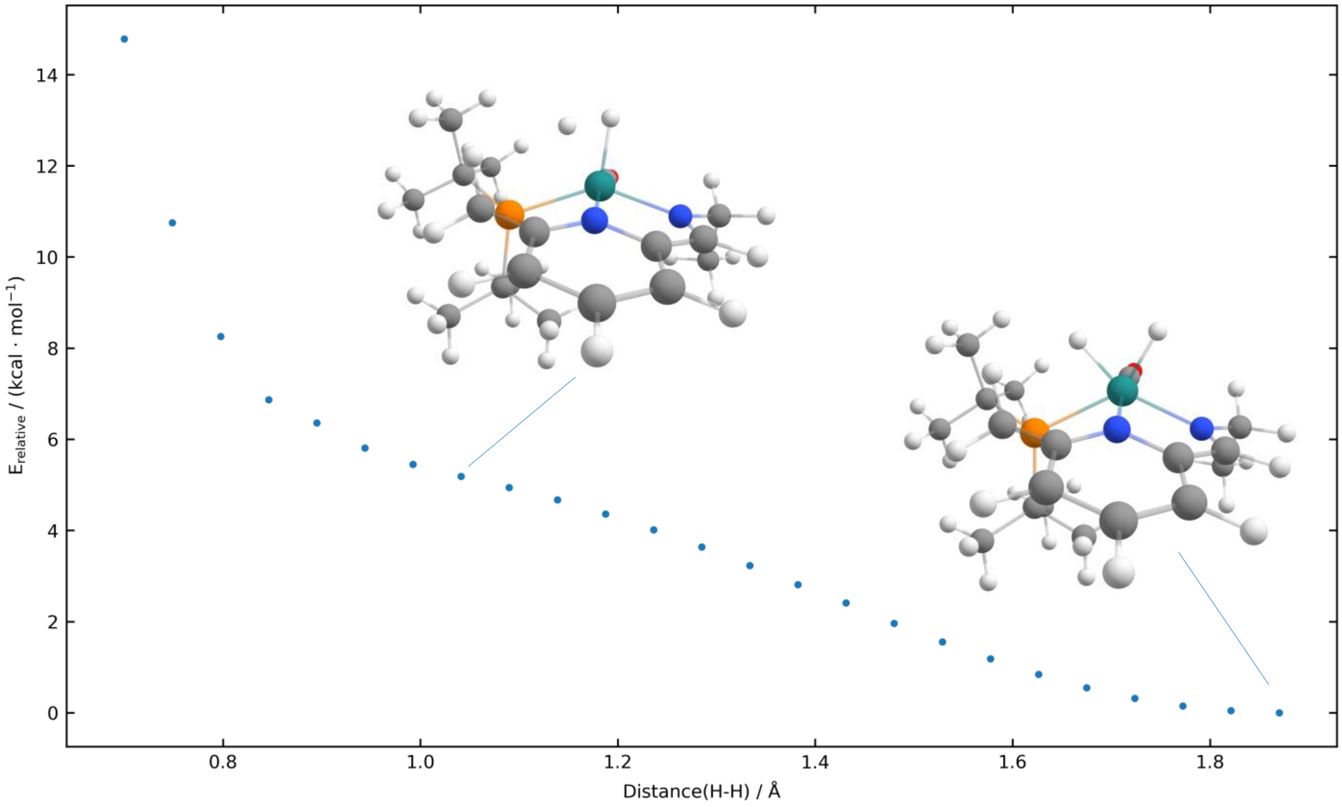


**Figure S5.** Relaxed scan for the reductive elimination of $H_2$ from **b**.

### Alternative Pathway: Initial addition of $H_2$ across Ru and C

Figure S6 shows an alternative pathway where $H_2$ initially adds to the Ru center and the imine carbon. This pathway proceeds with a much higher barrier than the minimum-energy pathway in Figure 7.

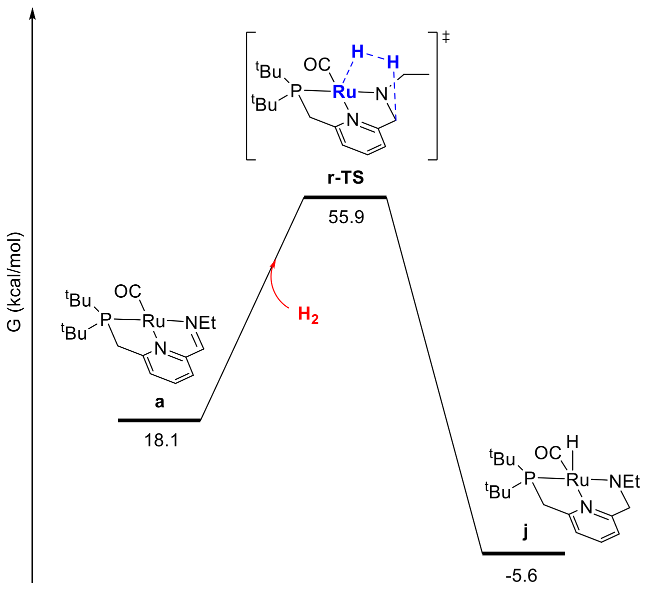


**Figure S6.** Initial addition of $H_2$ across the ruthenium center and the imine carbon.

### Alternative Pathway: Transfer of hydrogen from Ru to the imine carbon

Figure S7 shows an alternative pathway where a ruthenium hydride transfers to the imine carbon, rather than the imine nitrogen as occurs in the minimum-energy pathway in Figure 7. This pathway proceeds with a higher barrier than the minimum-energy pathway.

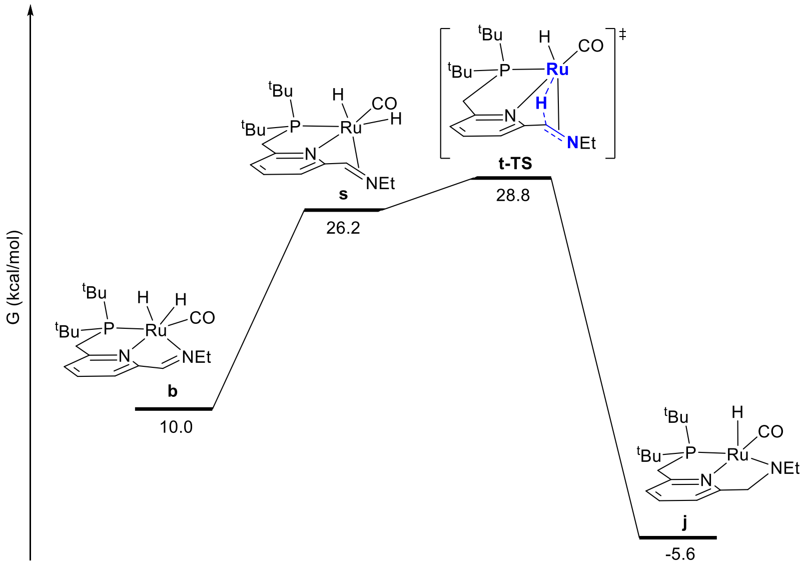


**Figure S7.** Ruthenium hydride migration to the imine carbon rather than the imine nitrogen.

## Alternative Pathway: Product Ru-NH transfers hydrogen to intermediate a instead of c

Figures S8 and S9 show two diastereomers of an alternative pathway where the product **Ru-NH** transfers hydrogen to intermediate **a** instead of **c**. These pathways proceed with a higher barrier than the minimum-energy pathway shown in Figure 8, and can also be excluded based on the kinetic data, since they incorporate hydrogen after the rate-determining step and would be expected to follow zero-order kinetics in the hydrogen pressure.

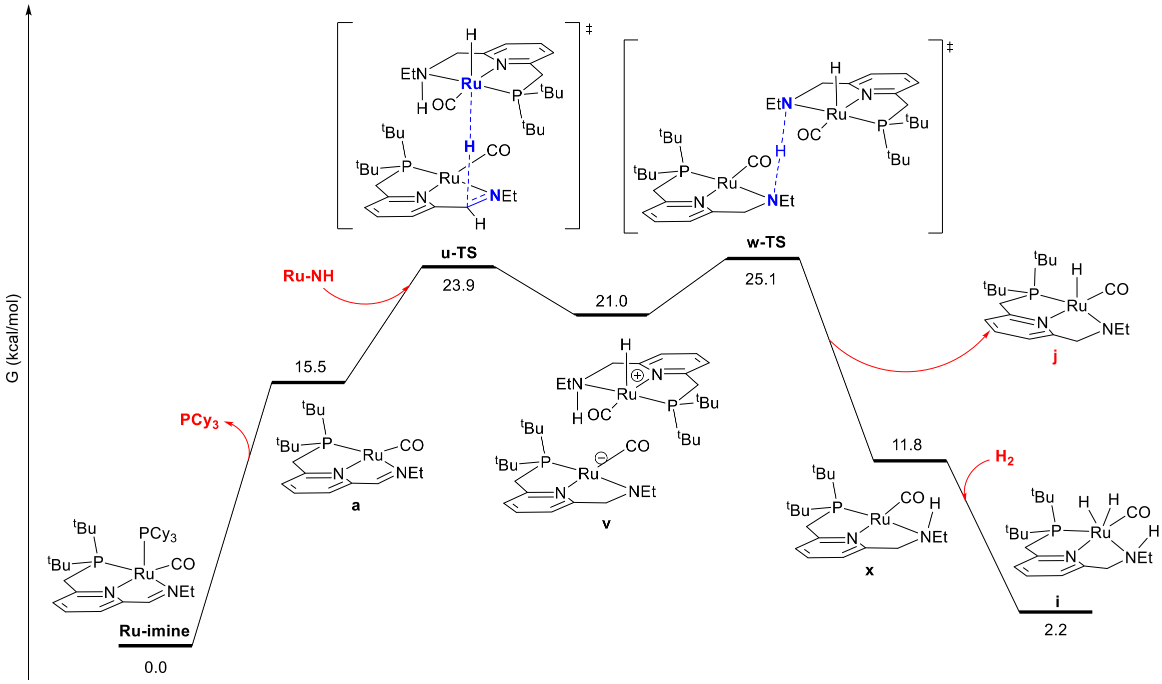


**Figure S8.** Product-mediated hydrogenation of **a** instead of **c**.

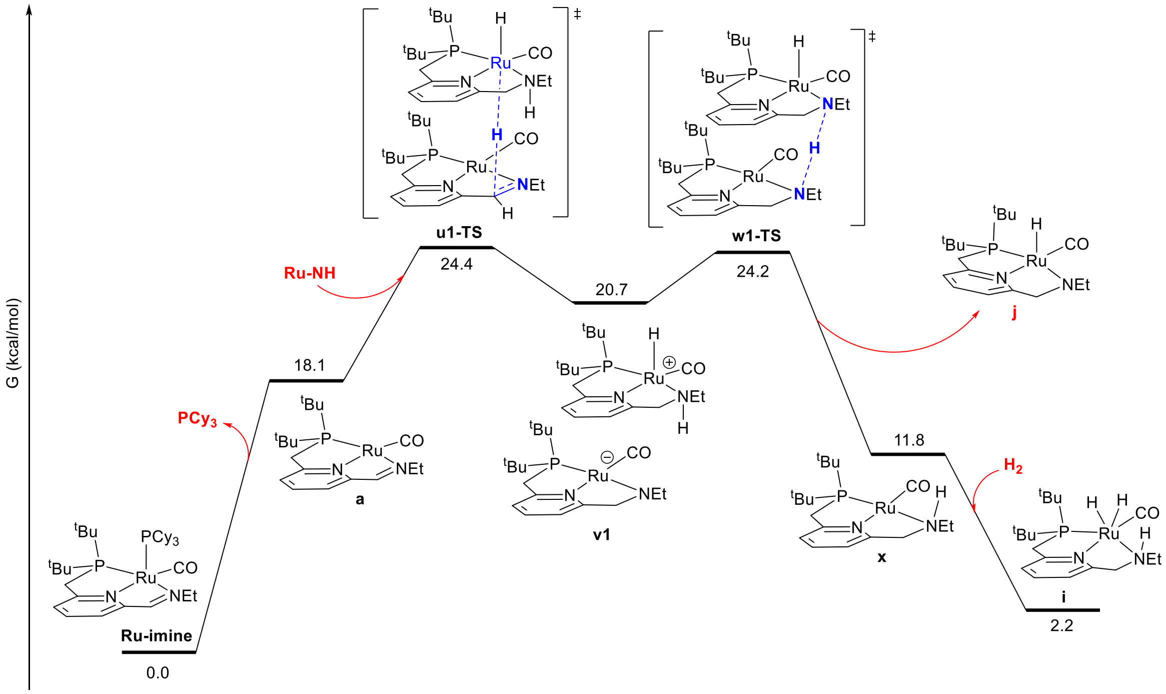


**Figure S9.** Product-mediated hydrogenation of **a** instead of **c**, diastereomeric pathway

**Alternative Pathway: Product Ru-NH transfers hydride to the imine nitrogen**

Figure S10 shows an alternative pathway where the product **Ru-NH** transfers a hydride to the imine nitrogen in **b** rather than the imine carbon. This pathway has a significantly higher barrier than the minimum-energy pathway shown in Figure 8.

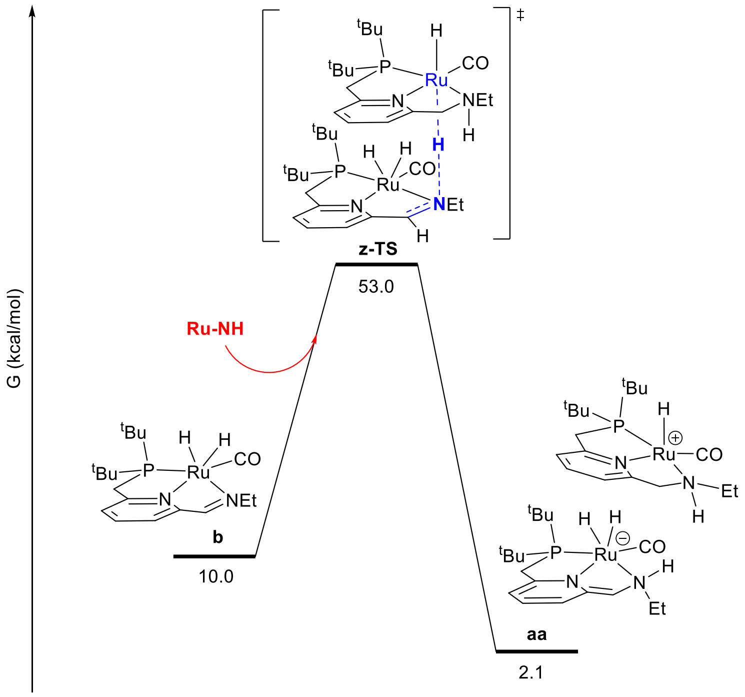


**Figure S10.** Hydride transfer to the imine nitrogen in **b** rather than the imine carbon.

**Alternative Pathway: Product Ru-NH protonates ruthenium instead of nitrogen**

Figures S11 and S12 show two diastereomeric versions of pathways where the product **Ru-NH** protonates the ruthenium center instead of the imine nitrogen, following hydride transfer to the imine carbon. Both pathways have higher barriers than the minimum-energy pathway shown in Figure 8.

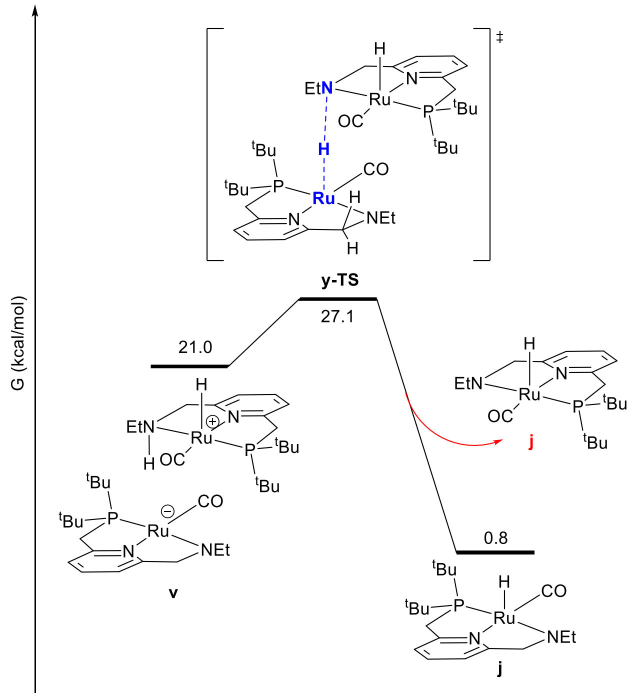


**Figure S11.** Proton transfer to Ru instead of N, first diastereomeric pathway.

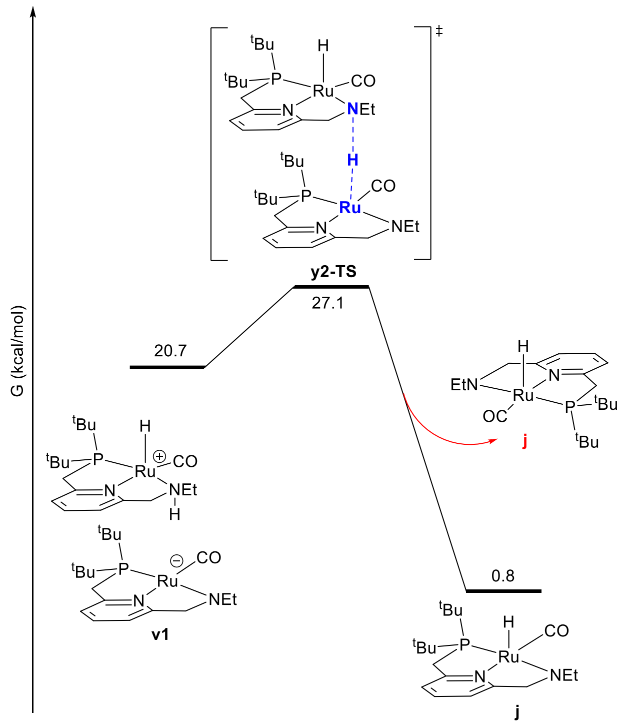


**Figure S12.** Proton transfer to Ru instead of N, second diastereomeric pathway.

**Alternative Pathway: Diastereomeric versions of the minimum-energy pathway shown in Figure 8**

Figures S13, S14, and S15 below show three diastereomeric versions of the hydride transfer in the minimum-energy pathway shown in Figure 8. In Figure S13, the opposite enantiomer of the product **Ru-NH** reacts with the same enantiomer of intermediate **b**. In Figures S14 and S15, **Ru-NH** approaches from the "bottom", opposite the cis-dihydrides in **b**. All three pathways have a higher barrier than the minimum-energy pathway shown in Figure 8.

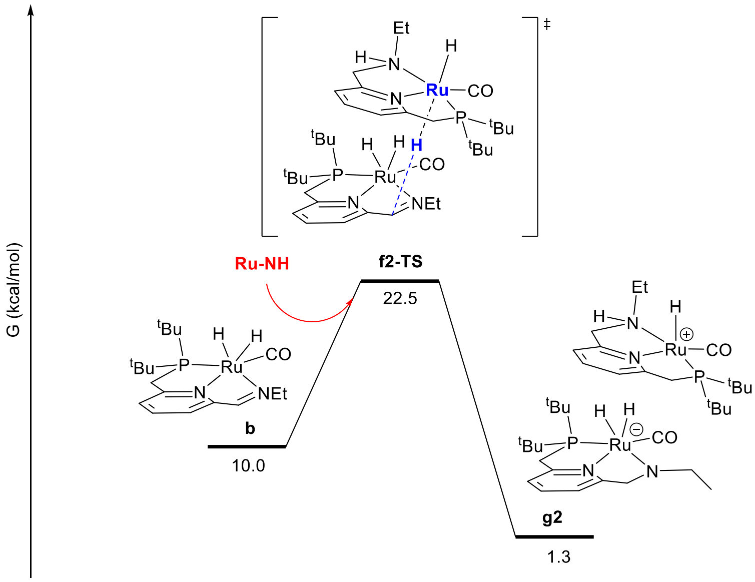


**Figure S13.** First diastereomeric pathway for hydride transfer to **b**.

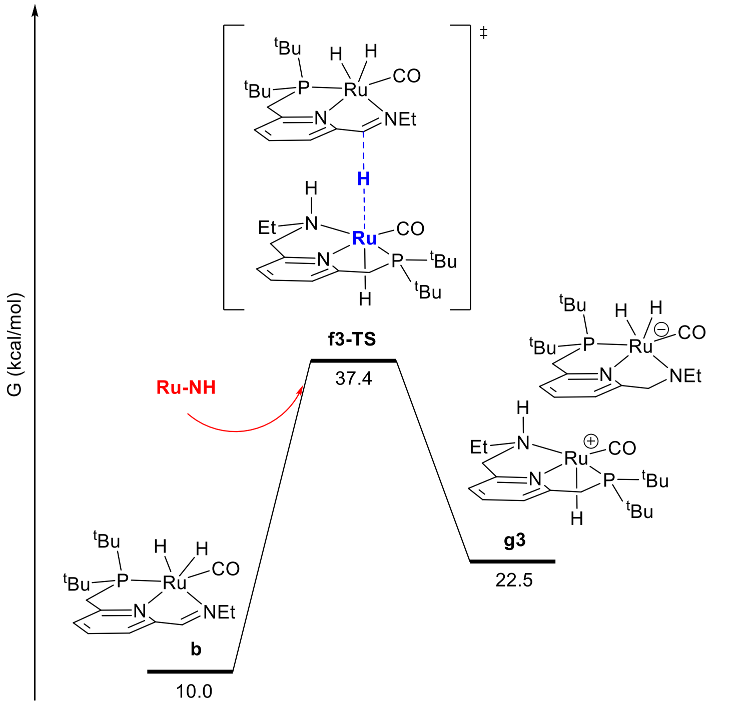


**Figure S14.** Second diastereomeric pathway for hydride transfer to **b**.

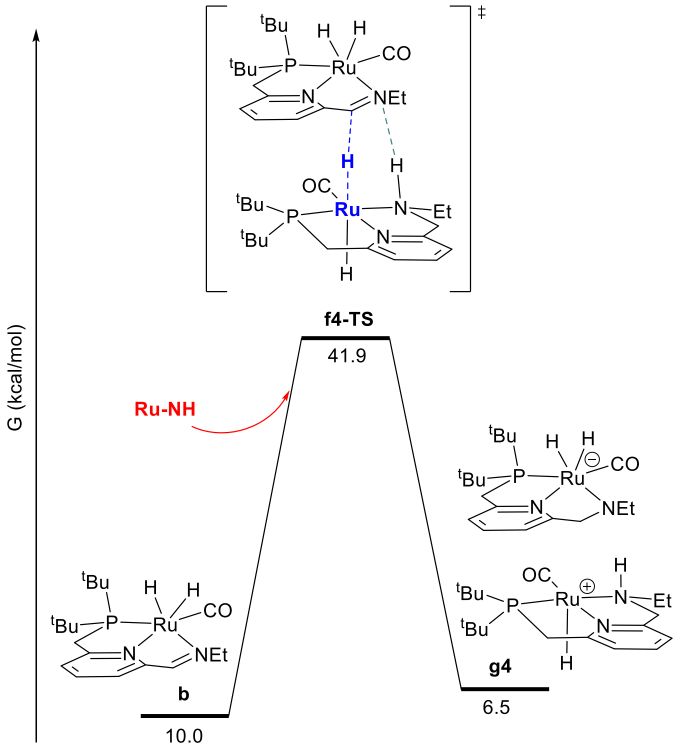


**Figure S15.** Third diastereomeric pathway for hydride transfer to **b**.

### Microkinetic Model

To test the agreement of the minimum-energy reaction network for the autocatalytic hydrogenation of **Ru-imine** to **Ru-NH** with the kinetic data, we constructed a microkinetic model using the free program COPASI[1], version 4.42 (Build 284). The molar concentration of $H_2$ was calculated using its known pressure-dependent solubility in toluene at 298.15 K.[2] The model incorporates every reaction in Figures 7-10, in both the forward and reverse directions. For most reactions, a transition state was identified: in these cases, rate constants were calculated using the Eyring equation, taking the transmission coefficient κ as equal to one:

$$k = \frac{\kappa k_B T}{h} e^{-\left(\frac{\Delta G^\ddagger}{RT}\right)}$$

For bimolecular reactions where a transition state was not located (1, 2, and 10), the free energy of the transition state was estimated by assuming the reaction in the downhill direction proceeds at the diffusion-controlled rate, as suggested by Harvey et al.[3] In toluene at 298.15 K, the diffusion-controlled second-order rate constant is $1.12 \times 10^{10}$ $M^{-1} \cdot s^{-1}$, which translates to an effective free-energy barrier of 3.7 kcal/mol. Table S2 shows the free-energy barriers, calculated from DFT, which were used as starting points in the kinetic model. In the table, values in **bold and red** represent "barrierless" bimolecular reactions modeled as diffusion-controlled.

**Table S2. Free-energy barriers used in the kinetic model**

| Number | Reaction | $\Delta G^{‡}_{fwd}$ (kcal/mol) | $\Delta G^{‡}_{rev}$ (kcal/mol) |
|---|---|---|---|
| 1 | Ru-imine ⇌ $PCy_3$ + a | **19.3** | **3.7** |
| 2 | a + $H_2$ ⇌ b | **3.7** | **9.3** |
| 3 | b ⇌ d | 15.2 | 22.1 |
| 4 | d + $H_2$ ⇌ Ru-NH | 13.3 | 28.3 |
| 5 | b + Ru-NH ⇌ g | 8.0 | 6.4 |
| 6 | g ⇌ i + j | 0.3 | 9.6 |
| 7 | i ⇌ l | 12.0 | 1.3 |
| 8 | l ⇌ n | 6.0 | 4.1 |
| 9 | n ⇌ Ru-NH | 3.0 | 23.4 |
| 10 | j + $H_2$ ⇌ p | **14.6** | **3.7** |
| 11 | p ⇌ Ru-NH | 7.5 | 24.7 |

To allow the optimization of individual intermediate or transition-state energies to obtain the best fit to the kinetic data, the COPASI model dynamically calculates the above barriers using the individual free energies for each species as $\Delta G^{‡} = G°_{TS} – G°_{reactant}$, as listed in Table S1. The rate constants are in turn calculated dynamically from the free-energy barriers. To obtain the fit to the kinetic data shown in Figure 11, we used the Parameter Estimation task in COPASI, allowing the energies of key, rate-determining intermediates and transition states to vary according to the Evolutionary Programming optimization algorithm. Our goal was to obtain the best fit achievable while adjusting the minimum number of free energies. We found that a very good fit was obtained by allowing the energies of only three species to vary: **a**, **c-TS**, and **f-TS**, which could not be improved by adjusting additional parameters. To verify that the adjusted free energies shown in Figure 11 (**a**, 17.506 ± 0.007; **c-TS**, 24.946 ± 0.012; and **f-TS**, 17.759 ± 0.004 kcal.mol) represented the global best fit, the Parameter Estimation task was repeated multiple times with randomized starting values, and was found to converge on the same result reliably.

The input data (copasi_input_trim_first_300_seconds.txt), COPASI model (microkinetic_model_trim_first_300_seconds.cps), and output showing the result of the fit (copasi_report_trim_first_300_seconds.txt) are included as Supporting Information. We elected to trim the first 300 seconds of data from each experiment, because the model did not reproduce the brief initial acceleration periods, which may be due to hydrogen uptake into solution at the start of the experiment. However, including this early data does not change the fitted values significantly. For comparison to the analysis using trimmed data, we have also included the complete input data (copasi_input_all_data.txt), COPASI model (microkinetic_model_all_data.cps), and COPASI output (copasi_report_all_data.txt) as Supporting Information. Figure S16 below shows the global fit obtained when the first 300 seconds of data are not trimmed, which may be compared to Figure 11 in the main text. The adjusted free energies (**a**, 17.650 ± 0.005; **c-TS**, 25.075 ± 0.014; and **f-TS**, 17.711 ± 0.004) are very close to those obtained by truncating the first 300 seconds of data from each experiment.

| Species | G(DFT) | G(kinetics) |
|---|---|---|
| **a** | 15.5 | 17.650 ± 0.005 |
| **c-TS** | 25.2 | 25.075 ± 0.014 |
| **f-TS** | 18.0 | 17.711 ± 0.004 |

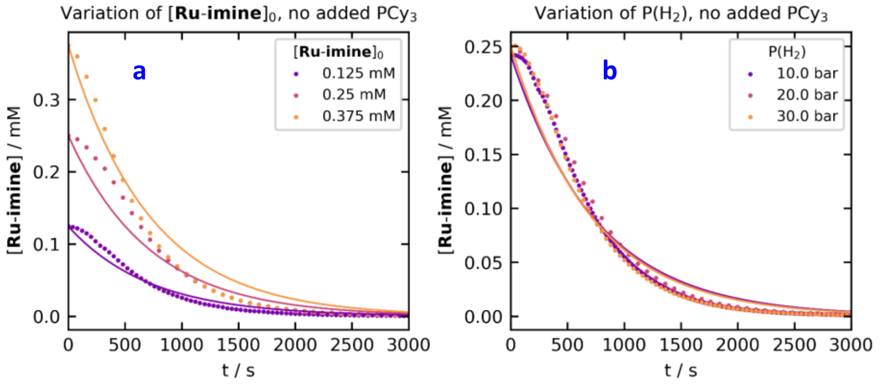


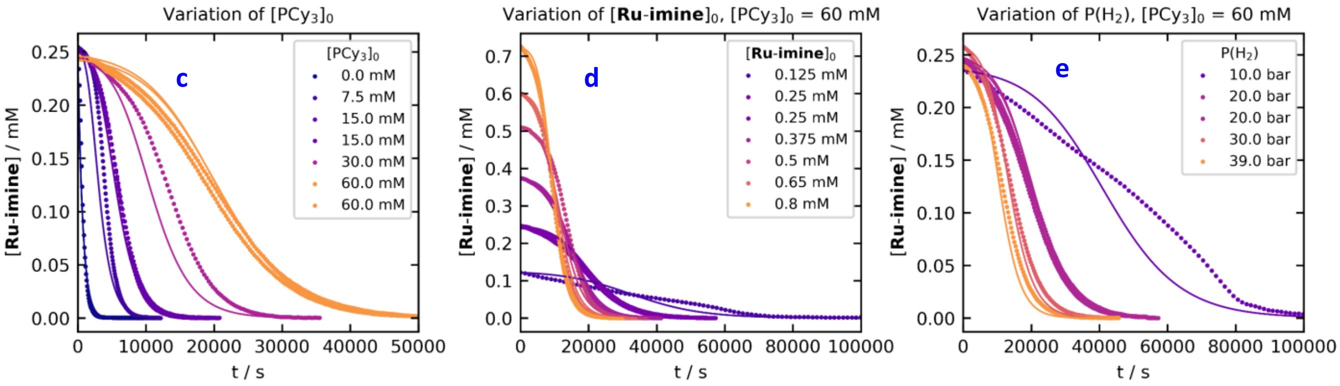


**Figure S16.** Global fit of the complete, non-trimmed kinetic data to the microkinetic model, after adjusting the free energies of **a**, **c-TS**, and **f-TS** to obtain the best fit (Compare with Figure 11, which shows the fit excluding data from the first 300 seconds of each experiment.)

**Analysis of the spin multiplicities of species calculated by DFT**

Although even-electron second-row transition-metal species are typically expected to have singlet ground states, we examined the possibility that the triplet state might be energetically competitive on the suggestion of a reviewer. In particular, structures involving electron-rich, low-coordinate ruthenium(0) might be expected to have an unusually small ligand field for a second-row transition-metal complex. To compare the singlet and triplet free energies using the DFT method applied in this work, we re-optimized each singlet structure as a triplet, using the same method as described in the Computational Methods section. Table S3 below shows the energies calculated for triplets. All columns are calculated in the same manner as in Table S1, and the rightmost column **ΔG(t – s)** represents the difference in the calculated free energies of the triplet and singlet structure.

As Table S3 shows, for most of the calculated structures the triplet is significantly higher in energy, indicating that the reaction pathway proceeds primarily through the singlet spin state. Some species, such as **k-TS**, **m-TS**, and **o-TS**, were not successfully optimized as triplets in an initial attempt, and significant effort was not expended to locate them as the adjacent triplet intermediates were significantly higher energy than the corresponding singlets. Species **s**, where the imine double bond is pi-complexed to Ru (see Figure S7), relaxed to **b** when optimized as a triplet. Species **z-TS**, representing a very high-energy alternative pathway on the singlet surface (see Figure S10), optimized as a ruthenium(0) $H_2$ sigma complex on the triplet surface, rather than the ruthenium(II) dihydride complex located on the singlet surface.

## Table S3. Energies of triplet structures calculated by DFT

| | E(opt) | E(single point) | G(corr) | Imag. | G(kcal) | Mass balance | G(tot) | G(rel) | ΔG(t - s) |
|---|---|---|---|---|---|---|---|---|---|
| **Direct Hydrogenation MEP (Fig. 7)** | | | | | | | | | |
| Ru-imine | -2371.303045 | -2372.065069 | 0.850119 | | -1487956.83 | 2 $H_2$ | -1489430.54 | 9.2 | 9.2 |
| a | -1324.306312 | -1324.679299 | 0.385675 | | -831004.27 | 2 $H_2$ + $PCy_3$ | -1489425.93 | 13.8 | -1.8 |
| a-H2 | -1325.47601 | -1325.857355 | 0.399003 | | -831735.15 | $H_2$ + $PCy_3$ | -1489419.95 | 19.7 | ---- |
| a-b-TS | -1325.467289 | -1325.846326 | 0.398730 | | -831728.40 | $H_2$ + $PCy_3$ | -1489413.20 | 26.5 | ---- |
| b | -1325.468392 | -1325.847434 | 0.400642 | | -831727.90 | $H_2$ + $PCy_3$ | -1489412.70 | 27.0 | 17.0 |
| c-TS | -1325.438288 | -1325.811024 | 0.398567 | -1222.65 | -831706.35 | $H_2$ + $PCy_3$ | -1489391.15 | 48.5 | 23.4 |
| d | -1325.459262 | -1325.830162 | 0.403445 | | -831715.30 | $H_2$ + $PCy_3$ | -1489400.10 | 39.6 | 36.6 |
| e-TS | -1326.588003 | -1326.963345 | 0.414774 | -1739.38 | -832419.27 | $PCy_3$ | -1489367.22 | 72.5 | 56.1 |
| Ru-NH | -1326.638698 | -1327.027488 | 0.423620 | | -832453.97 | $PCy_3$ | -1489401.92 | 37.8 | 49.8 |
| **Autocatalytic MEP (Figs. 8 and 9)** | | | | | | | | | |
| Ru-imine | -2371.303045 | -2372.065069 | 0.850119 | | -1487956.83 | 2 $H_2$ + Ru-NH | -2321934.28 | 9.2 | 9.2 |
| a | -1324.306312 | -1324.679299 | 0.385675 | | -831004.27 | 2 $H_2$ + Ru-NH + $PCy_3$ | -2321929.67 | 13.8 | -1.8 |
| b-sigma-complex | -1325.47601 | -1325.857355 | 0.399003 | | -831735.15 | $H_2$ + Ru-NH + $PCy_3$ | -2321923.69 | 19.7 | ---- |
| b-ox-add-TS | -1325.467289 | -1325.846326 | 0.398730 | | -831728.40 | $H_2$ + Ru-NH + $PCy_3$ | -2321916.94 | 26.5 | ---- |
| b | -1325.468392 | -1325.847434 | 0.400642 | | -831727.90 | $H_2$ + Ru-NH + $PCy_3$ | -2321916.44 | 27.0 | 17.0 |
| f-TS | -2652.192244 | -2652.956403 | 0.857555 | -523.95 | -1664214.00 | $H_2$ + $PCy_3$ | -2321898.80 | 44.6 | 26.7 |
| g | -2652.208931 | -2652.975662 | 0.862380 | | -1664223.06 | $H_2$ + $PCy_3$ | -2321907.86 | 35.6 | 24.0 |
| h-TS | -2652.18857 | -2652.956515 | 0.857706 | -858.21 | -1664213.98 | $H_2$ + $PCy_3$ | -2321898.78 | 44.7 | 32.9 |
| i | -1326.645452 | -1327.036529 | 0.423662 | | -832459.62 | $H_2$ + $PCy_3$ + j | -2321904.91 | 38.5 | 36.3 |
| k-TS | ---- | ---- | ---- | ---- | ---- | ---- | ---- | ---- | ---- |
| l | -1326.635556 | -1327.025107 | 0.420959 | | -832454.15 | $H_2$ + $PCy_3$ + j | -2321899.43 | 44.0 | 31.1 |
| m-TS | ---- | ---- | ---- | ---- | ---- | ---- | ---- | ---- | ---- |
| n | -1326.645329 | -1327.040666 | 0.421433 | | -832463.62 | $H_2$ + $PCy_3$ + j | -2321908.90 | 34.5 | 19.7 |
| o-TS | ---- | ---- | ---- | ---- | ---- | ---- | ---- | ---- | ---- |
| Ru-NH | -1326.638698 | -1327.027488 | 0.423620 | | -832453.97 | $H_2$ + $PCy_3$ + j | -2321899.26 | 44.2 | 49.8 |
| **Rehydrogenation of j (Fig. 10)** | | | | | | | | | |
| j | -1325.470092 | -1325.842169 | 0.402461 | | -831723.45 | $H_2$ + i + $PCy_3$ | -2321904.20 | 39.2 | 37.0 |
| p | -1326.642375 | -1327.019743 | 0.412987 | | -832455.79 | i + $PCy_3$ | -2321899.68 | 43.7 | 30.7 |
| q-TS | -1326.588178 | -1326.971599 | 0.424950 | -1701.28 | -832418.07 | i + $PCy_3$ | -2321861.96 | 81.5 | 61.0 |
| Ru-NH | -1326.638698 | -1327.027488 | 0.423620 | | -832453.97 | i + $PCy_3$ | -2321897.87 | 45.6 | 49.8 |
| **Initial addition of $H_2$ across Ru and C (Fig. S6)** | | | | | | | | | |
| a | -1324.306312 | -1324.679299 | 0.385675 | | -831004.27 | 2 $H_2$ + $PCy_3$ | -1489425.93 | 13.8 | -1.8 |
| r-TS | -1325.426253 | -1325.792874 | 0.398078 | -1680.04 | -831695.27 | $H_2$ + $PCy_3$ | -1489380.07 | 59.6 | 3.7 |
| j | -1325.470092 | -1325.842169 | 0.402461 | | -831723.45 | $H_2$ + $PCy_3$ | -1489408.25 | 31.4 | 37.0 |
| **Transfer of hydrogen from Ru to the imine carbon (Fig. S7)** | | | | | | | | | |
| b | -1325.468392 | -1325.847434 | 0.400642 | | -831727.90 | $H_2$ + $PCy_3$ | -1489412.70 | 27.0 | 17.0 |
| s | ---- | ---- | ---- | ---- | ---- | ---- | ---- | ---- | ---- |
| t-TS | -1325.424354 | -1325.793762 | 0.399032 | -1163.19 | -831695.23 | $H_2$ + $PCy_3$ | -1489380.03 | 59.7 | 30.9 |
| j | -1325.470092 | -1325.842169 | 0.402461 | | -831723.45 | $H_2$ + $PCy_3$ | -1489408.25 | 31.4 | 37.0 |

## Table S3 continued. Energies of triplet structures calculated by DFT

| | E(opt) | E(single point) | G(corr) | Imag. | G(kcal) | Mass balance | G(tot) | G(rel) | ΔG(t - s) |
|---|---|---|---|---|---|---|---|---|---|
| **Product Ru-NH transfers hydrogen to intermediate a instead of b (Fig. S8)** | | | | | | | | | |
| Ru-imine | -2371.303045 | -2372.065069 | 0.850119 | | -1487956.83 | 2 $H_2$ + Ru-NH | -2321934.28 | 9.2 | 9.2 |
| a | -1324.306312 | -1324.679299 | 0.385675 | | -831004.27 | 2 $H_2$ + Ru-NH + $PCy_3$ | -2321929.67 | 13.8 | -1.8 |
| u-TS | -2651.022276 | -2651.781479 | 0.839583 | -794.61 | -1663488.00 | 2 $H_2$ + $PCy_3$ | -2321909.66 | 33.8 | 9.9 |
| v | -2651.031708 | -2651.794078 | 0.843779 | | -1663493.28 | 2 $H_2$ + $PCy_3$ | -2321914.93 | 28.5 | 7.5 |
| w-TS | -2651.015791 | -2651.772708 | 0.839255 | -992.04 | -1663482.71 | 2 $H_2$ + $PCy_3$ | -2321904.36 | 39.1 | 14.0 |
| x | -1325.477392 | -1325.862438 | 0.408806 | | -831732.19 | 2 $H_2$ + $PCy_3$ + j | -2321914.33 | 29.1 | 17.3 |
| i | -1326.645452 | -1327.036529 | 0.423662 | | -832459.62 | $H_2$ + $PCy_3$ + j | -2321904.91 | 38.5 | 36.3 |
| **Product Ru-NH transfers hydrogen to intermediate a instead of b (Fig. S9)** | | | | | | | | | |
| Ru-imine | -2371.303045 | -2372.065069 | 0.850119 | | -1487956.83 | 2 $H_2$ + Ru-NH | -2321934.28 | 9.2 | 9.2 |
| a | -1324.306312 | -1324.679299 | 0.385675 | | -831004.27 | 2 $H_2$ + Ru-NH + $PCy_3$ | -2321929.67 | 13.8 | -1.8 |
| u1-TS | -2651.02266 | -2651.783289 | 0.838884 | -673.25 | -1663489.58 | 2 $H_2$ + $PCy_3$ | -2321911.24 | 32.2 | 7.8 |
| v1 | -2651.032597 | -2651.793095 | 0.843113 | | -1663493.08 | 2 $H_2$ + $PCy_3$ | -2321914.74 | 28.7 | 8.0 |
| w1-TS | -2651.014929 | -2651.770902 | 0.841475 | -1027.01 | -1663480.18 | 2 $H_2$ + $PCy_3$ | -2321901.84 | 41.6 | 17.4 |
| x | -1325.477392 | -1325.862438 | 0.408806 | | -831732.19 | 2 $H_2$ + $PCy_3$ + j | -2321914.33 | 29.1 | 17.3 |
| i | -1326.645452 | -1327.036529 | 0.423662 | | -832459.62 | $H_2$ + $PCy_3$ + j | -2321904.91 | 38.5 | 36.3 |
| **Product Ru-NH transfers hydrogen to imine nitrogen in b instead of imine carbon (Fig. S10)** | | | | | | | | | |
| b | -1325.468392 | -1325.847434 | 0.400642 | | -831727.90 | $H_2$ + Ru-NH + $PCy_3$ | -2321916.44 | 27.0 | 17.0 |
| z-TS | -2652.175667 | -2652.939708 | 0.852775 | -1383.8 | -1664206.53 | $H_2$ + $PCy_3$ | -2321891.33 | 52.1 | -0.9 |
| aa | -2652.193741 | -2652.956719 | 0.859151 | | -1664213.20 | $H_2$ + $PCy_3$ | -2321898.00 | 45.4 | 43.3 |
| **Product Ru-NH protonates ruthenium instead of nitrogen (Fig. S11)** | | | | | | | | | |
| v | -2651.031708 | -2651.794078 | 0.843779 | | -1663493.28 | 2 $H_2$ + $PCy_3$ | -2321914.93 | 28.5 | 7.5 |
| y-TS | -2650.99166 | -2651.736998 | 0.836286 | -730.75 | -1663462.16 | 2 $H_2$ + $PCy_3$ | -2321883.82 | 59.6 | 32.5 |
| j | -1325.470092 | -1325.842169 | 0.402461 | | -831723.45 | 2 $H_2$ + $PCy_3$ + j | -2321905.59 | 37.8 | 37.0 |
| **Product Ru-NH protonates ruthenium instead of nitrogen (Fig. S12)** | | | | | | | | | |
| v1 | -2651.032597 | -2651.793095 | 0.843113 | | -1663493.08 | 2 $H_2$ + $PCy_3$ | -2321914.74 | 28.7 | 8.0 |
| y2-TS | -2650.992022 | -2651.741982 | 0.836110 | -205.97 | -1663465.40 | 2 $H_2$ + $PCy_3$ | -2321887.06 | 56.4 | 28.6 |
| j | -1325.470092 | -1325.842169 | 0.402461 | | -831723.45 | 2 $H_2$ + $PCy_3$ + j | -2321905.59 | 37.8 | 37.0 |
| **Diastereomeric version of the MEP shown in Figure 8 (Fig. S13)** | | | | | | | | | |
| b | -1325.468392 | -1325.847434 | 0.400642 | | -831727.90 | $H_2$ + Ru-NH + $PCy_3$ | -2321916.44 | 27.0 | 17.0 |
| f2-TS | -2652.188536 | -2652.953785 | 0.853462 | -681.73 | -1664214.93 | $H_2$ + $PCy_3$ | -2321899.73 | 43.7 | 21.2 |
| g2 | -2652.209708 | -2652.968911 | 0.864143 | | -1664217.72 | $H_2$ + $PCy_3$ | -2321902.52 | 40.9 | 39.6 |
| **Diastereomeric version of the MEP shown in Figure 8 (Fig. S14)** | | | | | | | | | |
| b | -1325.468392 | -1325.847434 | 0.400642 | | -831727.90 | $H_2$ + Ru-NH + $PCy_3$ | -2321916.44 | 27.0 | 17.0 |
| f3-TS | -2652.175611 | -2652.940057 | 0.853994 | -783.65 | -1664205.98 | $H_2$ + $PCy_3$ | -2321890.78 | 52.7 | 15.2 |
| g3 | -2652.201357 | -2652.971212 | 0.858624 | | -1664222.62 | $H_2$ + $PCy_3$ | -2321907.43 | 36.0 | 13.5 |
| **Diastereomeric version of the MEP shown in Figure 8 (Fig. S15)** | | | | | | | | | |
| b | -1325.468392 | -1325.847434 | 0.400642 | | -831727.90 | $H_2$ + Ru-NH + $PCy_3$ | -2321916.44 | 27.0 | 17.0 |
| f4-TS | -2652.173231 | -2652.936603 | 0.853777 | -1019.73 | -1664203.95 | $H_2$ + $PCy_3$ | -2321888.75 | 54.7 | 12.7 |
| g4 | -2652.193085 | -2652.962844 | 0.857545 | | -1664218.05 | $H_2$ + $PCy_3$ | -2321902.85 | 40.6 | 34.1 |

Although the coordination of $H_2$ to **a** and its subsequent oxidative addition to give **b** is barrierless on the singlet electronic potential energy surface (see Figure S5), we were able to locate the $H_2$ σ-complex **a-H2** and the oxidative-addition transition state **a-b-TS** on the triplet surface. Figure S17 below compares the singlet and triplet pathways for the conversion of **Ru-imine** to **b** by loss of $PCy_3$ and addition of $H_2$. Although **Ru-imine** and **b** have lower-energy singlet configurations, the triplet state is preferred by 1.7 kcal/mol for the 16-electron ruthenium(0) complex **a** at the ωB97X-D3/def2-QZVPPD//r$^2$SCAN-3c level of theory. Clearly, the reaction of **a** with $H_2$ to give **b** must proceed on the singlet surface, as the oxidative addition transition state **a-b-TS** and the product ruthenium(II) dihydride **b** are much higher in energy on the triplet surface. However, if the calculated energies are accurate, it is conceivable that the dissociation of $PCy_3$ from **Ru-imine** to give **a** might involve crossing to the triplet state along the dissociation pathway, which might result in a slightly lower barrier for the initial $PCy_3$ dissociation.

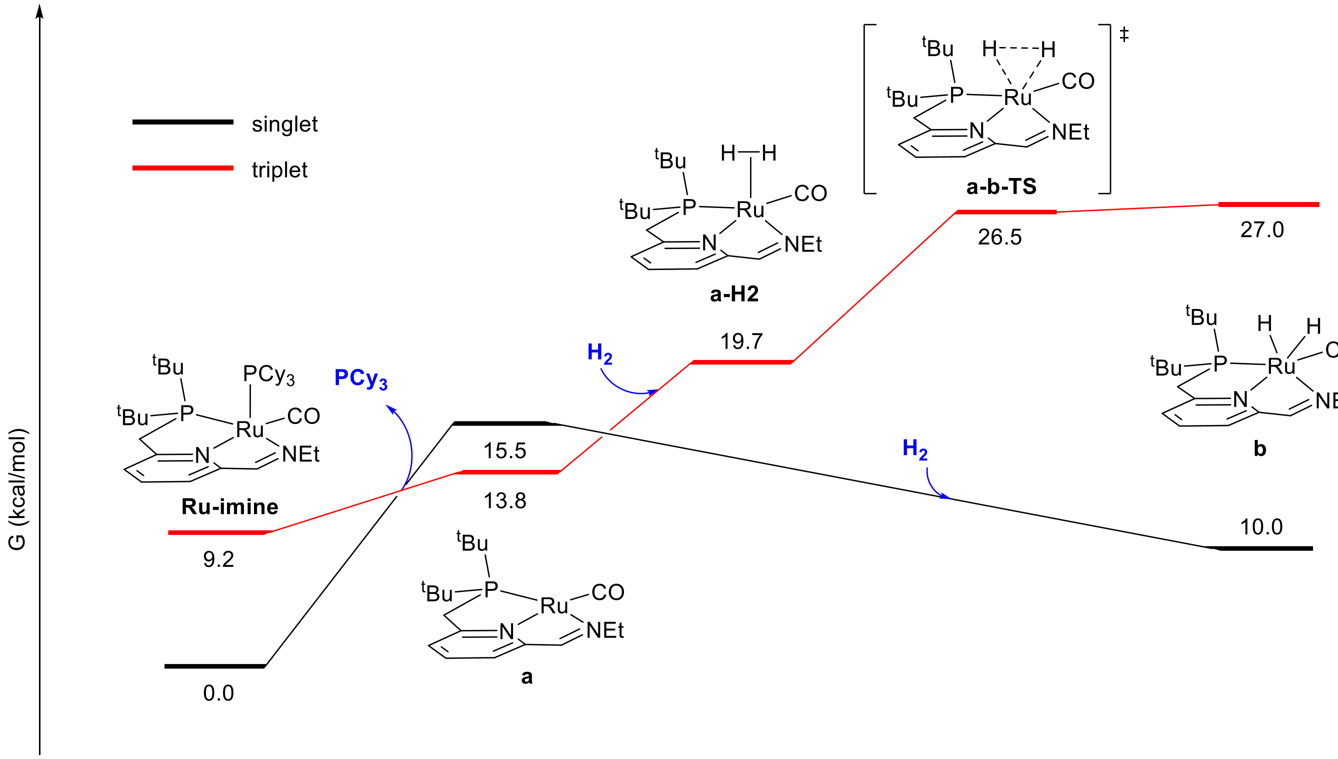


**Figure S17.** Comparison of the singlet (black) and triplet (red) free energy surfaces for the reaction of **Ru-imine** with $H_2$ to produce **b** and $PCy_3$, as calculated at the ωB97X-D3/def2-QZVPPD//r$^2$SCAN-3c level of theory.

Because the relative free energy of **a** along the conversion of **Ru-imine** to **b** is partially rate-determining in the kinetic analysis, we sought to further assess the accuracy of this calculation. The calculation of singlet-triplet energy gaps is particularly challenging for density functional theory. Functionals such as the range-separated hybrid ωB97X-D3, which are well-suited for accurate thermochemistry in closed-shell, single-reference systems, may overestimate the relative stability of higher-spin configurations.[4]

First, we calculated single-point energies for the species **Ru-imine**, **a**, and **b** using a range of dispersion-corrected density functionals that span the levels of Perdew's "Jacob's ladder," selected for their high accuracy in a recent benchmark study of single-reference, open-shell reactions of transition-metal complexes.[5] Table S4 shows singlet-triplet gaps calculated for these species using the GGA functional PBE-D4, meta-GGA r$^2$SCAN-D3, hybrid TPSS0-D4,

and range-separated hybrid ωB97X-D3, all with the def2-QZVPPD basis set and the SMD(toluene) solvent model. The calculated singlet-triplet gaps span 10 kcal/mol for **Ru-imine**, 9.2 kcal/mol for **a**, and 6.7 kcal/mol for **b**. These results emphasize the difficulty for DFT in accurately identifying the ground state for complexes such as **a**, where the singlet-triplet gap is small.

| Species | PBE-D4 | $r^2$SCAN-D3 | TPSS0-D4 | ωB97X-D3 |
|---|---|---|---|---|
| **Ru-imine** | 19.2 | 16.4 | 12.3 | 9.2 |
| **a** | 7.4 | 2.1[a] | -2.7[a] | -1.8[a] |
| **b** | 23.7 | 21.7 | 19.4 | 17.0 |

**Table S4.** Singlet-triplet free-energy gaps ($G_{triplet}$ – $G_{singlet}$) in kcal/mol for three species using four density functionals. [a]For these functionals, **a** optimized as an open-shell singlet.

As **a** was calculated to have a small energy difference separating the singlet and triplet states, this complex is likely to exhibit multi-reference character and may be inadequately described by single-reference DFT methods. As a further probe into the possible multi-reference character of **a**, we calculated the finite-temperature DFT based fractional occupation number weighted electron density (FOD) to estimate the level of static correlation in **a**, using the default method in ORCA 6.0.0 (TPSS/def2-TZVP, T = 5000 K).[6] The high $N_{FOD}$ value of 1.23 calculated for the open-shell singlet state indicates the potential for multi-reference character. An FOD plot with an isosurface value of 0.005 $e^-/bohr^3$ is shown below in Figure S18. As **a** shows a significant FOD, delocalized between the ruthenium center and the pyridine-imine fragment of the pincer ligand, multi-reference character is likely and the spin-state energetics calculated by DFT should be treated with caution. Because the relative energy of the triplet state of **a** is not expected to be reliably calculated by DFT, and because the kinetic data supports a free energy for **a** of 17.5 kcal/mol, close to the calculated free energy for the singlet of 15.5 kcal/mol, we focus on the singlet species in the main text (Figures 7 and 8).

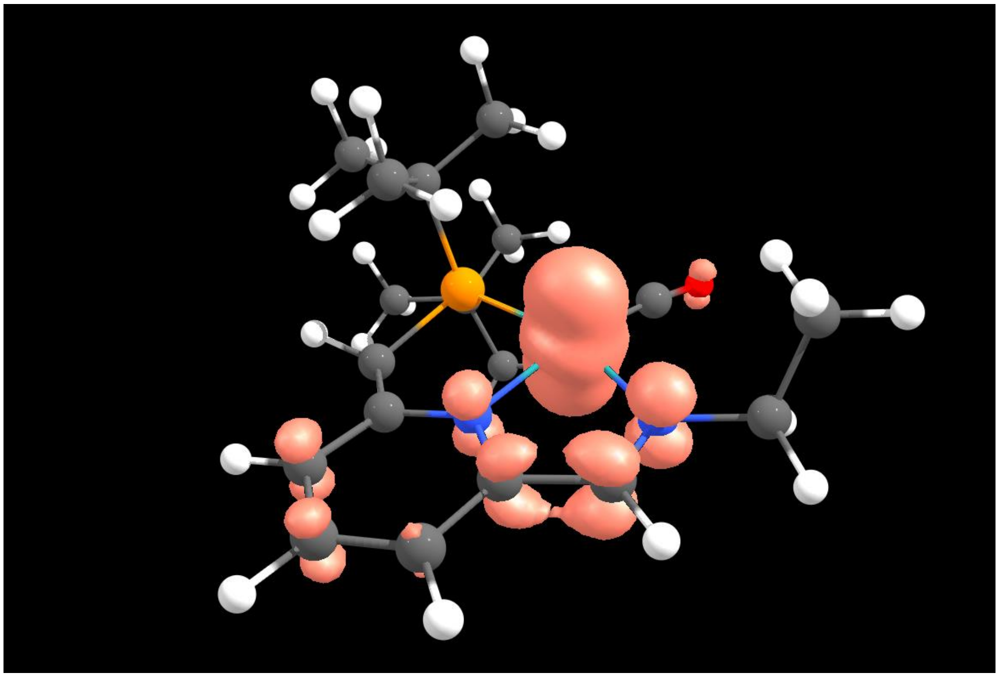

**Figure S18.** FOD plot for **a** with an isosurface value of 0.005 $e^-/bohr^3$.

## References


(1) Hoops, S.; Sahle, S.; Gauges, R.; Lee, C.; Pahle, J.; Simus, N.; Singhal, M.; Xu, L.; Mendes, P.; Kummer, U. Copasi—a Complex Pathway Simulator. *Bioinformatics* **2006,** *22*, 3067-3074.

(2) Brunner, E. Solubility of Hydrogen in 10 Organic Solvents at 298.15, 323.15, and 373.15 K. *J. Chem. Eng. Data* **1985,** *30*, 269-273.

(3) Harvey, J. N.; Himo, F.; Maseras, F.; Perrin, L. Scope and Challenge of Computational Methods for Studying Mechanism and Reactivity in Homogeneous Catalysis. *ACS Catal.* **2019,** *9*, 6803-6813.

(4) (a) Mortensen, S. R.; Kepp, K. P. Spin Propensities of Octahedral Complexes from Density Functional Theory. *J Phys Chem A* **2015,** *119*, 4041-50. (b) Morgante, P.; Peverati, R. Comparison of the Performance of Density Functional Methods for the Description of Spin States and Binding Energies of Porphyrins. *Molecules* **2023,** *28*.

(5) Maurer, L. R.; Bursch, M.; Grimme, S.; Hansen, A. Assessing Density Functional Theory for Chemically Relevant Open-Shell Transition Metal Reactions. *J. Chem. Theory Comput.* **2021,** *17*, 6134-6151.

(6) Grimme, S.; Hansen, A. A Practicable Real-Space Measure and Visualization of Static Electron-Correlation Effects. *Angew. Chem. Int. Ed.* **2015,** *54*, 12308-12313.
